# Enhancing Relationship-Centered Communication and Feedback in Emergency Medicine Through Applied Improvisation (EM-PROV)

**DOI:** 10.5070/M5.52345

**Published:** 2026-04-30

**Authors:** Jordan Valentin, Abbas Husain, Brendan Freeman

**Affiliations:** * Northwell Health & Staten Island University Hosptial, Department of Emergency Medicine, Staten Island, NY

## Abstract

**Audience:**

This small group session is intended for emergency medicine residents, medical students, and faculty.

**Introduction:**

Improvisational techniques offer a novel and effective approach to teaching relationship-centered communication (RCC) and enhancing learner feedback in emergency medicine (EM).^1^ Improvisational theater (improv) is a form of spontaneous performance where all things or most things are made up on the spot. The “yes, and” principle—accepting a partner’s idea (“yes”) and building upon it (“and”)—reflects core improv values such as affirmation, spontaneity, active listening, and empathy, all of which contribute to psychologically safe learning environments.^1^ It is an engaging practice that can foster creativity, build confidence, and enhance communication and social skills. Improv helps participants become more adaptable, attuned to emotional tone, and comfortable with uncertainty. These are critical elements of high-quality interpersonal feedback.^2^,^3^,^4^

In the fast-paced EM setting, where teaching and supervision often occur in real time, the ability to deliver concise, respectful, and actionable feedback is essential. Improv-based learning provides a low-stakes space to explore tone, content, and delivery without fear of error. Prior studies demonstrate that improv improves communication performance, team collaboration, and confidence in difficult conversations.^5^,^6^ This module builds on that foundation by integrating structured feedback models and core RCC principles with improvisational exercises, allowing participants to refine skills through play, reflection, and peer interaction. As EM continues to emphasize communication and professionalism milestones, improv offers a compelling adjunct to traditional faculty or resident development by combining emotional literacy, interpersonal skills, and educational theory in a single interactive format.

**Educational Objectives:**

By the end of this session, learners will be able to improve relationship-centered communication (RCC): 1) define “yes, and” and its role in RCC, and 2) demonstrate active listening and responsiveness using improvisational techniques such as “yes, and,” gift-giving, establishing scene, and callbacks. They will also be able to improve learner feedback: 1) define “yes, and” and its role in learner feedback, 2) review three evidence-based feedback models through a “yes, and” lens, and 3) practice improv techniques and deliver structured feedback in real-time peer scenarios using improvisational techniques such as “yes, and,” gift-giving, establishing scenes, and callbacks.

**Educational Methods:**

Using Kern’s six-step approach, this curriculum was designed to address gaps in relationship-centered communication (RCC) and feedback skills among emergency medicine residents.^7^,^8^,^9^,^10^ The first two workshops focused on RCC, linking improvisational principles to patient communication through the Three-Function Model, which emphasizes building relationships, understanding the patient’s perspective, and collaborating on care decisions. Sessions incorporated facilitator discussions, a video clip from Whose Line Is It Anyway? and small-group improv exercises. The third workshop targeted feedback skills for Graduate Medical Education (GME) leadership, introducing concepts such as “yes, and,” credible feedback, and evidence-based models, and included interactive activities like the “Red Ball” exercise and improv-based learner scenarios to reinforce effective communication and feedback practices.

**Research Methods:**

A post-intervention survey with a 5-point Likert scale was administered immediately after all three sessions via Google Forms. Participants were asked about the structure of the activity, length, engagement, relevance to practice, and the facilitator’s skill. Open-response questions included: “Describe a specific moment during the improv session that stood out to you.” “How did it impact your understanding or approach to feedback or communication?” “How do you see the skills learned today translating to your future clinical practice?” “How likely are you to use “yes, and” in your communication with colleagues and patients?” “What challenges did you encounter when applying improv techniques during this session?” Thematic analysis of reflective prompts was performed.

**Results:**

Fifty-two learners participated and responded to the post-intervention survey. This included a 5-point Likert scale and open-response questions administered for all three sessions. Respondents rated highly (4 or 5 on Likert scale) the activity structure (96.1%), length (84.3%), engagement (100%), relevance to practice (92.3%), and facilitator skill (88.5%). Thematic analysis of free-text answers revealed themes of “enjoyment/engagement,” “connection to patient care,” “applying improv to feedback,” and “openness to future application.”

**Discussion:**

This applied improvisation curriculum provided an effective modality to practice RCC and feedback skills among EM learners and faculty. High engagement, relevance to practice, and strong facilitator impact highlight this effectiveness. The thematic analysis findings underscore the value of connecting improv techniques to patient care and feedback delivery.

**Topics:**

Relationship-centered communication, feedback, applied improvisation.

## USER GUIDE

**Table:** 

List of Resources:	
Abstract	36
User Guide	38
Small Groups Learning Materials	41
Appendix A: PowerPoint	52
Appendix B: Video Clip Link	53

**Learner Audience:**

Medical Students, Emergency Medicine Residents, Faculty

**Time Required for Implementation:**

The activity lasted one hour but could be shorter or longer depending on the number of learners in the program and the activities included.

**Recommended Number of Learners Per Instructor:**

4–5

**Topics:**

Relationship-centered communication, feedback, applied improvisation.

**Objectives:**

By the end of this session, learners will be able to:

- Define “yes, and” and its role in RCC.
- Demonstrate active listening and responsiveness using improvisational techniques such as “yes, and,” gift-giving, establishing scene, and callbacks.

Learner Feedback

- Define “yes, and” and its role in learner feedback.
- Review three evidence-based feedback models through a “yes, and” lens.
- Practice improv techniques and deliver structured feedback in real-time peer scenarios using improvisational techniques such as “yes, and,” gift-giving, establishing scenes, and callbacks.

### Linked objectives and methods

This format was chosen because it allows the learner to be an active participant, observe the function of improv and “yes, and” through demonstration, and then practice the skills in a simulated environment with real-time feedback.

#### RCC

The objective: Defining “yes, and” and its role in RCC links to the introductory section and video debrief, where “yes, and” is introduced and illustrated as a tool for validating patient perspectives and building authentic rapport. The objective: Demonstrating active listening and responsiveness using improvisational techniques such as “yes, and,” gift-giving, establishing scene, callbacks, and open-ended questions links to the video debrief, facilitator demo, and resident improv activity, where participants observe and apply these techniques in real-time. The printed handout “Pearls” reinforces these principles with clinical parallels.

#### Feedback

The objective: Defining “yes, and” and its role in learner feedback links to the introductory slides and Red Ball activity, where participants explore foundational concepts of listening, miscommunication, and validation, setting the stage for feedback conversations. The objective: Reviewing three evidence-based feedback models through a “yes, and” lens” links to the presentation of Self-Assessment Feedback/Facts Encouragement Direction (SFED), Ask, Tell, Ask (ATA), and Relationship Reaction Content Coaching (R2C2) models in the slide deck and the Red Ball debrief, which draws attention to standardizing communication using feedback structures.^13^,^14^,^15^ The objective: Practicing improv techniques and delivering structured feedback in real-time peer scenarios using improvisational techniques such as “yes, and,” gift-giving, establishing scene, callbacks, and open-ended questions” links to the small group learner feedback role-play scenarios, where participants practice feedback delivery in emotionally charged or challenging situations, applying improv principles within a structured educational context.

### Recommended pre-reading for facilitator

Consider watching episodes of “Whose Line Is It Anyway?” to become familiar with the format.^12^ The references provided in this manuscript provide a useful foundation as well.

### Optional Learner Responsible Content (LRC)

To familiarize yourself with the format, consider watching episodes of “Whose Line Is It Anyway?”^11^

### Small group application exercise (sGAE)

See the following attached materials for this small group exercise

- Appendix A: PowerPoint slides used for the feedback session.
- Appendix B: Video clip link to guide your discussion during the RCC workshop.

### Results and Tips for Successful Implementation

Fifty-two learners participated in this study. Respondents rated (4 or 5 on Likert scale) the activity structure highly (96.1%), and on length (84.3%), on engagement (100%), on relevance to practice (92.3%), and on facilitator skill (88.5%). Qualitative data and themes are summarized below.

1. **Describe a specific moment during the improv session that stood out to you. How did it impact your understanding or approach to feedback or communication?**
  Participants highlighted how improv fostered active listening, curiosity, and relational awareness during feedback. Simple, engaging activities made core feedback principles more tangible, and the session helped validate common challenges in giving feedback.
  *Participant A: “Was actually easier to do the feedback in this setting b/c you were truly curious… had to listen to how the situation plays out.”*
  *Participant B: “I liked the red ball activity because it was simple but highlighted a lot of important points in how to give feedback.”*
2. **How do you see the skills learned today translating to your future clinical practice?**
  Participants plan to apply improv principles, especially “yes, and” and active listening to enhance feedback and communication. Many noted intentions to be more deliberate, empathetic, and structured in future feedback sessions, with some aiming to integrate these techniques into faculty development.
  *Participant C: “I will be mindful to announce I am giving feedback and will encourage my faculty to do so. I will use “yes, and” in giving.”*
3. **How likely are you to use “yes, and” in your communication with colleagues and patients?**
  Most participants said they will likely use improv techniques like “yes, and” in clinical communication. Common reasons included its value in fostering validation, open dialogue, and effective feedback. Some noted it would be especially useful in mentoring and team interactions, while a few felt usage would depend on context.
  *Participant D: “Very likely because the analogy is useful.”*
  *Participant E: “I’ll use it because it helps promote an open communication of acceptance.”*
4. **What challenges did you encounter when applying improv techniques during this session?**
  Challenges included time constraints, group dynamics with unfamiliar peers, and noise distractions. A few noted the limits of “yes, and,” suggesting it may not fit every scenario. Others felt more structured facilitation could improve the experience. Several participants, however, reported no significant challenges.
  *Participant F: “I think communicating with people we may not know well and figuring out how to develop a relationship quickly to make the scenario work.”*
  *Participant G: “‘Yes, and...’ has its limitations. Implement ‘yes, but...’ once in a while.”*

### Representative Constructive Feedback

Participants praised the session as creative, engaging, and impactful, especially for its connection between improv, listening, and feedback. Many found it a fun and memorable learning experience, noting its potential as an icebreaker or intern orientation tool.

*Participant J: “The build from improv skills to active listening to feedback skills was clever and very effective.”*

*Participant K: “Loved the activity… reminded me the importance of listening. Like really leaning in and listening.”*

Some participants wanted more explicit practice of “yes, and” via demonstrations by facilitators and concrete examples or phrases for real-world application.

*Participant H: “Could be more specific about how to apply “yes, and”‘ to feedback. A role play by the facilitators would be helpful.”*

*Participant I: “Structure great. Would appreciate practical sentences one can use in giving feedback.”*

As a result of the feedback, the authors added more facilitator demonstration of feedback and communication scenarios, structured the debrief around these themes, and provided attendees with the “Pearls” document below summarizing how the improv principles explored relate to patient care, communication, and feedback.

This study was conducted in a limited number of academic EM programs with voluntary participation. Self-reported confidence improvements may not reflect sustained behavioral change in clinical settings, and qualitative reflections are subject to bias. Future studies should examine longer-term outcomes and assess whether repeated improv exposure improves actual patient communication or feedback effectiveness as rated by peers or learners.

## Supplementary Information



## Figures and Tables

**Figure:**
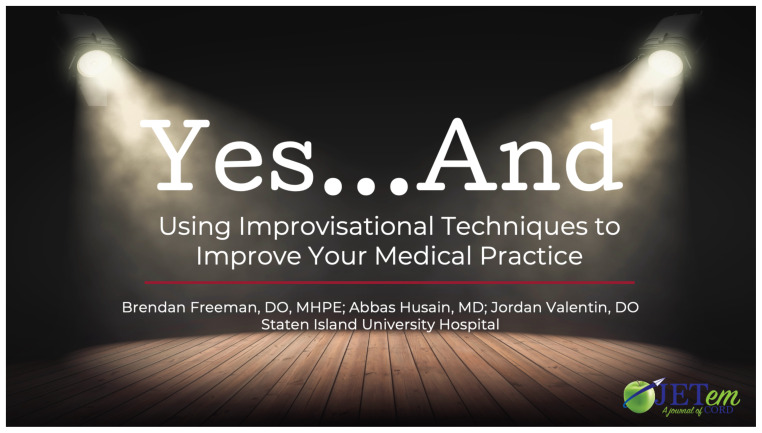
Please see associated PowerPoint file

## SMALL GROUPS LEARNING MATERIALS

### Small Group Application Exercise (sGAE)

#### Session 1: Relationship-Centered Communication (RCC)

Total Time: ~30 minutes

1. Introduction (1 min)
  Introduce improvisation. Emphasize how improv is used in daily life. You can use the analogy of kids’ make-believe play to set the tone. Ask about everyone’s experience with improv. Establish relevance to clinical care and tie-back to events that happen in the ED. Highlight and define common improvisational techniques and rules. Use the “Pearls” worksheet to guide this discussion.
2. Show Video Example (3–5 min)
  Play this video for the group: https://youtu.be/ZHicGPV7Gcs?si=HP6cmpT0_VocwvCK. Ask the group to pay attention to moments of “yes, and,” gift-giving, callbacks, and establishing the scene. When done, ask them for initial impressions: what worked well in the scene, and what did not work well, and where they saw moments of improvisational techniques being used. As each named technique comes up, tie this back to patient care and communication. Use the “Pearls” sheet to guide the discussion.
3. Facilitator Demo Scene (5 min)
  Two facilitators act out a scene based on an audience suggestion (This can be literally anything! Encourage them to get creative. Clinical or nonclinical. The zanier, the better.) Debrief by identifying improvisational techniques used and link them back to clinical practice. Use the “Pearls” sheet to guide the discussion.
4. Resident Improv Activity (10 min)
  Groups of 2–3 work well for this portion. If you use three people, use a “freeze/tag-out” format where two improvisers start the scene and the third yells “freeze.” The improvisers will stop speaking and freeze in the position they are in. The third person can then select one of the improvisers to tag out and assume the physical position before beginning a new scene inspired by the position. Use suggestions from the audience to start the scene. As the facilitator, try to end the scenes around 2–3 minutes on a “high-note”-- a point in the scene that gets a large laugh from the group or seems like a good ending. Rotate through all groups until everyone has a chance to go. Participation is not required, but the facilitators find that learners feel more comfortable participating by modeling the scenes first. After each scene, the facilitator can provide suggestions to improve future scenes rooted in the improvisational techniques discussed on the “Pearls” sheet and occasionally emphasize how these relate to patient care.
5. Wrap-Up & Key Takeaways (5 min)
  Ask the group for any thoughts or impressions from the scenes. Emphasize validation, empathy, and communication throughout the scenes. Review the “Pearls” sheet. Ask each learner for one takeaway from the day.

#### Session 2: Learner Feedback

Total Time: ~50–55 minutes

1. Introduction & Objectives (15 min)
  Use slides to introduce “yes, and” in the context of feedback. Present SFED, ATA, and R2C2 models.
2. Red Ball Activity (5 min)
  In one large group, play a game of “Red Ball.” The facilitator should start this activity by asking participants to make a large circle in the center of the room. They should explain the game to the group. Red ball is played by passing an invisible “red ball” to one another. To pass it, a player makes eye contact with someone across the circle and says, “Red ball.” If the other person is ready, they reply, “Red ball, thank you,” and the ball is mimed being tossed and caught. As the game progresses, additional imaginary objects, each with unique characteristics like weight, size, or behavior can be introduced (eg, “spiky ball,” “cat,” “slow-motion ball”) by the facilitator, requiring players to adapt their body language and energy accordingly. The goal is not only to keep the imaginary objects moving smoothly but also to support ensemble connection and creative play. Play until the game falls apart–it will. Debrief on communication breakdowns and tie to feedback challenges. Some talking points may include: (1) not seeing someone throwing you something, (2) receiving a lot of throws at once, (3) did anyone throw someone else a ball in one way and then it got transformed into something else? We take in a lot of physical data as clinicians which can get misinterpreted as we pass it along to others. This is true with feedback. Using feedback models helps standardize this approach.
3. Facilitator Demo Scene (5 min)
  Facilitators perform a two-or-three-person scene, depending on number of facilitators, using an audience suggestion (can use “freeze/tag-out” from before if needed).
4. Small Group Improv + Feedback Scenarios (20 min)
  Divide into groups of 4–5 participants, each led by a facilitator and do two-or-three-person scene based on audience suggestions. Highlight moments of “yes, and” and connect back to feedback scenarios. As before, participation is not required but is encouraged.
5. Final Debrief (2 min)
  Ask participants what they will take into their teaching practice.

**Brief wrap-up:**

Appendix A: PowerPoint slides used for the feedback session.

Appendix B: Video clip to guide your discussion during the RCC workshop. https://youtu.be/ZHicGPV7Gcs?si=HP6cmpT0_VocwvCK

### RCC Pearls

#### Rule 1: Say “yes, and”!

*Accept the reality created by your partner and then build on it.*

What this means in improv:

In improv, performers create a scene together without a script. “Yes” means you accept whatever your partner just said as the shared reality. “And” means you add something to move the scene forward. It’s not about agreement; it’s about acceptance and contribution. If you deny your partner’s idea, the scene stalls.

RCC Translation:

In patient care, “yes” looks like validating emotion. “And” looks like curiosity and exploration. You accept the patient’s experience as real and then build on it.

Improv Example:

- *“Yes, the dragon chose us as its new masters... and it wants to open a smoothie bar downtown.”*

**RCC Function:** Connect

**Microskill Corollary 1 (Yes):** Express Empathy (Reflection, Legitimation, Exploration)

ED Example:

- *(Reflection) “You seem really anxious about what’s going on.”*
- *(Legitimization) “Most people would feel overwhelmed in your situation.”*
- *(Exploration) “Can you tell me more about what brought you in today?”*

**Microskill Corollary 2 (And):** Explore Perspectives (Ideas, Concerns, Expectations)

ED Example:

- *(Ideas) “What do you think is going on?”*
- *(Concerns) “What worries you most about this?”*
- *(Expectations) “What were you hoping we’d do for you today?”*

#### Rule 2: Don’t Block

*Avoid denying or undoing your partner’s contributions.*

What this means in improv:

Blocking happens when you negate, contradict, or ignore what your partner just offered. When you block, the scene loses momentum and trust. Good improv keeps the shared story intact.

RCC Translation:

In the ED, blocking can happen when we dismiss concerns, redirect too quickly, or let barriers (technology, interruptions) break connection. Instead, acknowledge the reality in the room and work within it.

Improv Example:

**Do this:**

- *Partner: “You forgot our anniversary again!”*
- *You: “I did...and I can’t believe I messed up after last year’s lava cake disaster.”*

**Not this:**

- *Partner: “You forgot our anniversary again!”*
- *You: “No I didn’t. That never happened.”*

**RCC Function:** Connect

**Microskill Corollary:** Acknowledge Technology and Barriers

ED Example:

- *“I know these monitors and alarms can be distracting. Let me close the curtain, and we can focus on your questions one at a time.”*

#### Rule 3: Establish the Location

*Scenes happen somewhere. Anchor the setting early.*

What this means in improv:

Improv scenes need grounding. Early details about where we are and who we are prevent confusion and create psychological safety for both performers and audience.

RCC Translation:

Patients feel safer when the clinical “scene” is anchored. Clear introductions, role clarification, and orientation reduce uncertainty and establish trust.

Improv Example:

- *“Grab your helmet! The troll tribunal meets in the cave behind the waterfall.”*

**RCC Function:** Connect

**Microskill:** Set the Stage

ED Example:

- *“Hello. I’m Dr. X. I’m the resident on the team taking care of you today. Can I see your wristband to confirm I’m talking to the right person?”*

#### Rule 4: Change!

*Characters must evolve.*

What this means in improv:

Scenes are dynamic. Characters evolve as new information emerges. If nothing changes, the scene feels flat. Good improv adapts to new developments.

RCC Translation:

Clinical reasoning evolves. When new data appears, we narrate that shift. Sharing changes in thinking helps patients feel included rather than confused by pivots.

Improv Example:

- *“When I left, you were a farmer with a dream. Now you’re Queen of the Sky Bees.”*

**RCC Function:** Collaborate

**Microskill:** Share Information

ED Example:

- *“Earlier we thought this might be pneumonia, but the chest X-ray looks normal. Let’s talk about next steps and what else could be going on.”*

#### Rule 5: Give Gifts

*Offer something that helps your partner shine or go deeper.*

What this means in improv:

A “gift” is an offer that makes your partner look interesting, capable, or dimensional. It gives them something meaningful to respond to and deepens the scene.

RCC Translation:

In patient encounters, gifts are thoughtful contributions that move beyond surface-level dialogue such as sharing your clinical concern transparently.

Improv Example:

- *“You always did keep that sword under your pillow ever since the goblin uprising. Maybe it’s time we finally use it.”*

**RCC Function:** Assess and Understand

**Microskill:** Add Your Concerns

ED:

- *“You’ve shared your concerns about the pain being muscular. One thing I’m wondering about, given your history, is whether your heart could be involved. I’d like to rule that out.”*

#### Rule 6: Use Callbacks

*Refer back to something from earlier to build connection and continuity.*

What this means in improv:

A callback references something established earlier in the scene. It creates continuity, rewards attention, and builds cohesion.

RCC Translation:

In medicine, callbacks reinforce understanding and partnership. Teach-back is a structured callback. It loops back to earlier information to confirm alignment.

Improv Example:

- *“Didn’t they warn us not to use the tunnel on the right? And now here you are, married to the mole king and planning the honeymoon in the fungus mines!”*

**RCC Function:** Collaborate

**Microskill:** Teach-Back

ED:

- *“To make sure I explained things clearly, can you walk me through how you’ll use the inhaler at home?”*

### Feedback Pearls

#### Rule 1: Say “Yes, and”!

*Accept the reality created by your partner and then build on it.*

What this means in improv:

You validate what your partner created before adding your contribution.

Feedback Translation:

“Yes” validates effort or emotion. “And” introduces growth. Without “yes,” feedback feels harsh. Without “and,” it feels incomplete.

Improv Example:

- *“Yes, the dragon chose us as its new masters... and it wants to open a smoothie bar downtown.”*

**Feedback Function:** Validate and Advance

**Microskill 1 (Yes):** Acknowledge emotion or effort

**Microskill 2 (And):** Offer direction, suggestion, or growth-oriented feedback

Teaching Example*:*

- *“You looked overwhelmed when the trauma came in. That’s understandable, and I think walking through the ABCs beforehand next time will help you feel more prepared.”*

#### Rule 2: Don’t Block

*Avoid denying or undoing your partner’s contributions.*

What this means in improv:

Blocking shuts down collaboration. It signals, “Your contribution doesn’t count.”

Feedback Translation:

When we deny challenges or minimize struggles, we erode trust. Normalizing difficulty keeps learners psychologically safe and open to improvement.

Improv Example:

**Do this:**

- *Partner: “You forgot our anniversary again!”*
- *You: “I did... and I can’t believe I messed up after last year’s lava cake disaster.”*

**Not this:**

- *Partner: “You forgot our anniversary again!”*
- *You: “No I didn’t. That never happened.”*

**Feedback Function:** Build Trust

**Microskill:** Normalize challenges and invite dialogue

Teaching Example:

- *“Many residents have trouble recognizing early sepsis. Let’s talk about the cues we missed and how to spot them faster next time.”*

#### Rule 3: Establish the Location

*Scenes happen somewhere. Anchor the setting early.*

What this means in improv:

Clear context reduces ambiguity.

Feedback Translation:

Announcing feedback creates safety. It prevents surprise critique and signals intentional coaching.

Improv Example:

- *“Grab your helmet! The troll tribunal meets in the cave behind the waterfall.”*

**Feedback Function:** Clarify Context

**Microskill:** Announce feedback or clarify intent

Teaching Example:

- *“I wanted to take a few minutes before sign-out to give you some feedback on how the lac repair went.”*

#### Rule 4: Change!

*Characters must evolve.*

What this means in improv:

Growth makes scenes compelling.

Feedback Translation:

Highlighting evolution reinforces a growth mindset. Learners need to see progress to stay motivated.

Improv Example:

- *“When I left, you were a farmer with a dream. Now you’re Queen of the Sky Bees.”*

**Function:** Foster a Growth Mindset

**Microskill:** Highlight progress, frame future goals

Teaching Example:

- *“You’ve really improved your presentations over the past few months. Let’s work on your assessment/plans next.”*

#### Rule 5: Give Gifts

*Offer something that helps your partner shine or go deeper.*

What this means in improv:

A gift elevates your partner and invites depth.

Feedback Translation:

When we name strengths and ask reflective questions, we empower learners to understand their own competence.

Improv Example:

- *“You always did keep that sword under your pillow ever since the goblin uprising. Maybe it’s time we finally use it.”*

**Feedback Function:** Encourage Reflection

**Microskill:** Acknowledge strengths, invite insight

Teaching Example:

- *“You managed that airway calmly, even with limited prep time. How did you stay focused?”*

#### Rule 6: Use Callbacks

*Refer back to something from earlier to build connection and continuity.*

What this means in improv:

Callbacks create narrative continuity.

Feedback Translation:

Referencing prior performance reinforces learning arcs and shows that improvement is noticed.

Improv Example:

- *“Didn’t they warn us not to use the tunnel on the right? And now here you are, married to the mole king and planning the honeymoon in the fungus mines!”*

**Feedback Function:** Foster a Growth Mindset

**Microskill:** Reference past feedback, link progress

*Teaching Example:*

- *“Last month you had your first STEMI case. I noticed today’s STEMI went much smoother. You picked it up on EKG right away!”*

## Appendix B Video clip to guide your discussion during the RCC workshop

https://youtu.be/ZHicGPV7Gcs?si=HP6cmpT0_VocwvCK
