# Supplementary material for: Enhancing Relationship-Centered Communication and Feedback in Emergency Medicine Through Applied Improvisation (EM-PROV)
Source: J Educ Teach Emerg Med. 2026 Apr 30;11(2):SG36–53. doi: 10.5070/M5.52345 (PMC13152378; doi:10.5070/M5.52345)
Supplement: Supplementary file 1 [file 11-2-SG36-Appendix_A.pptx]

## Slide 1
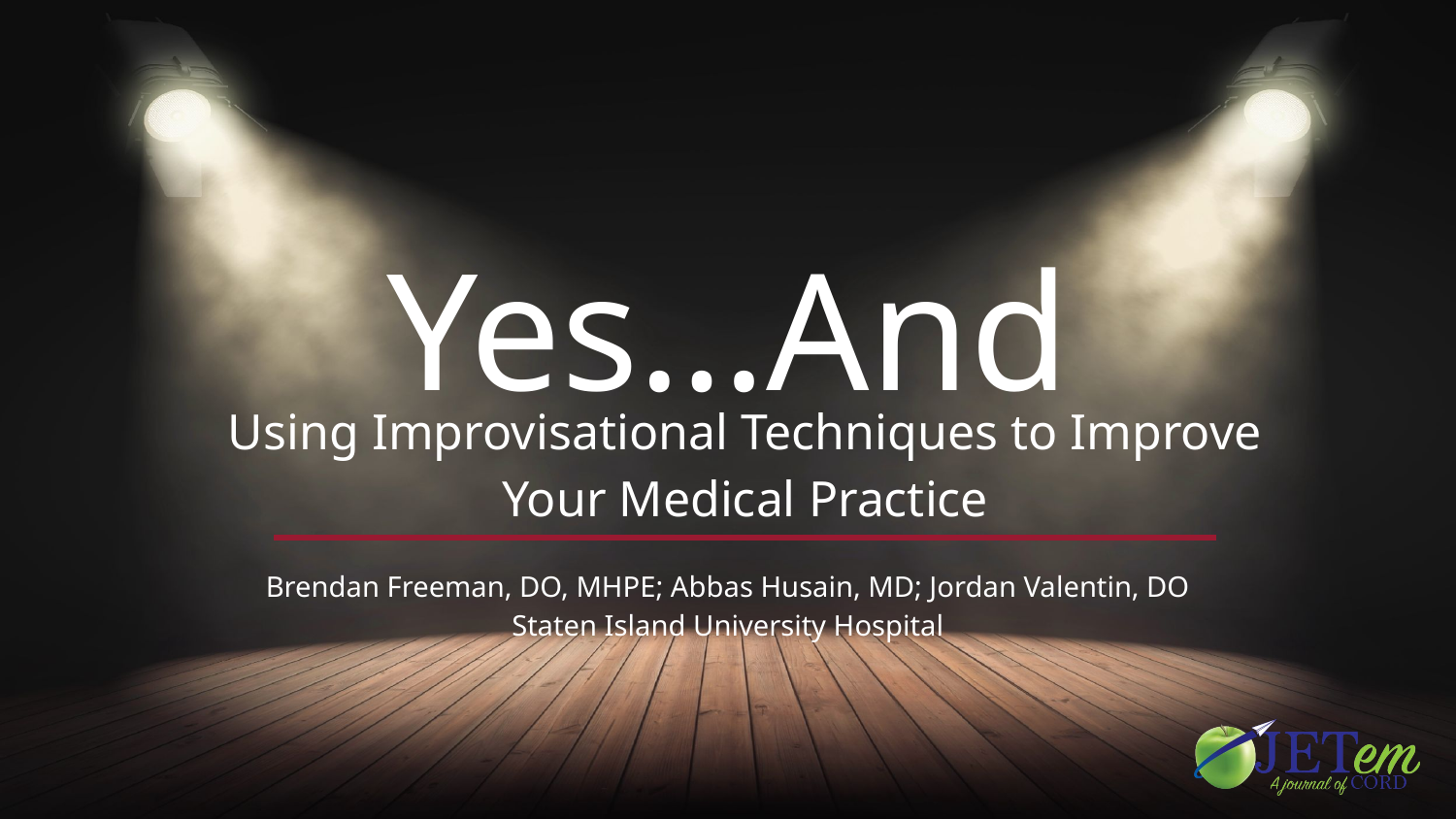

Yes…And
Using Improvisational Techniques to Improve Your Medical Practice
Brendan Freeman, DO, MHPE; Abbas Husain, MD; Jordan Valentin, DO
Staten Island University Hospital

## Slide 2
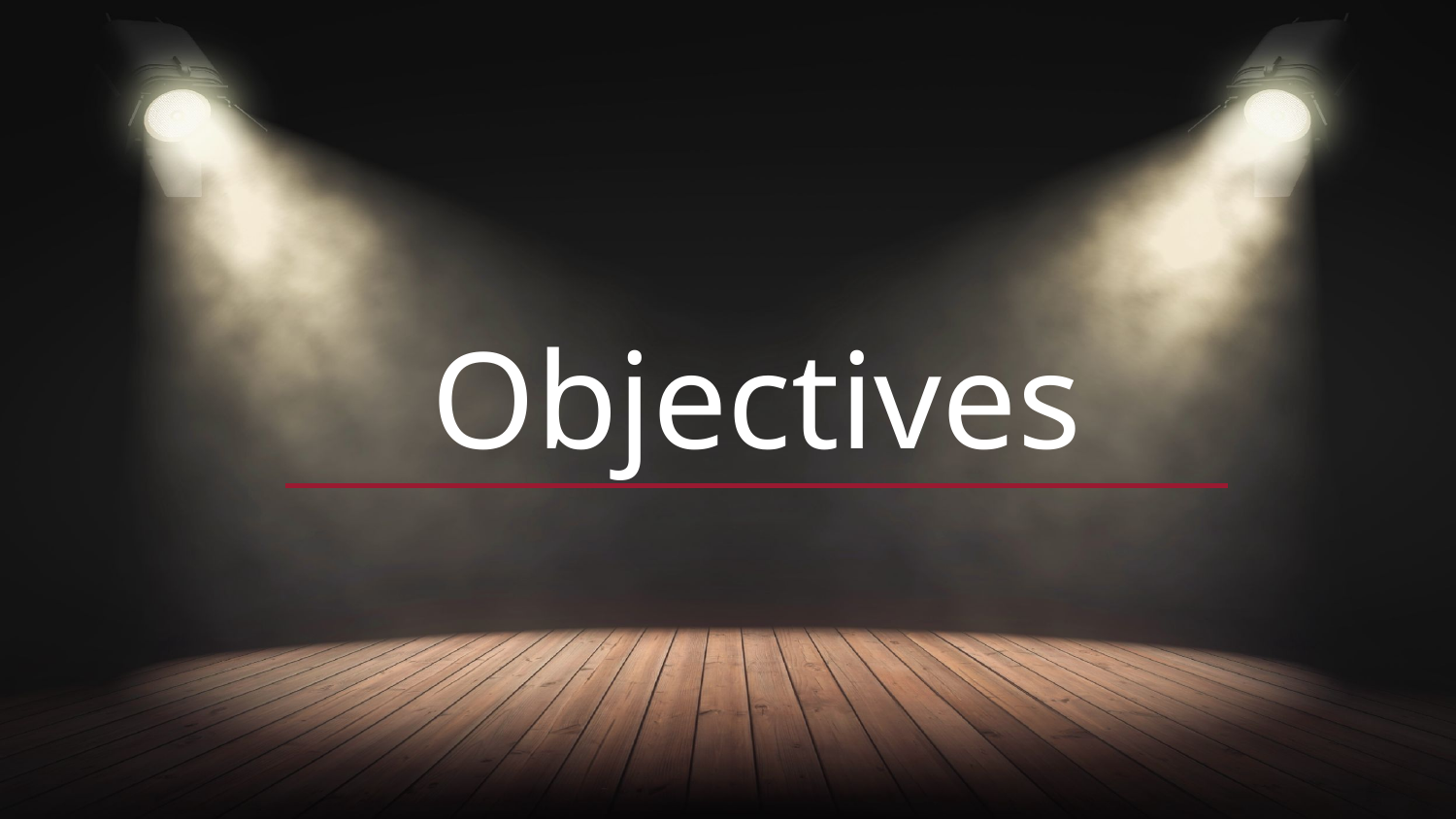

Objectives

## Slide 3
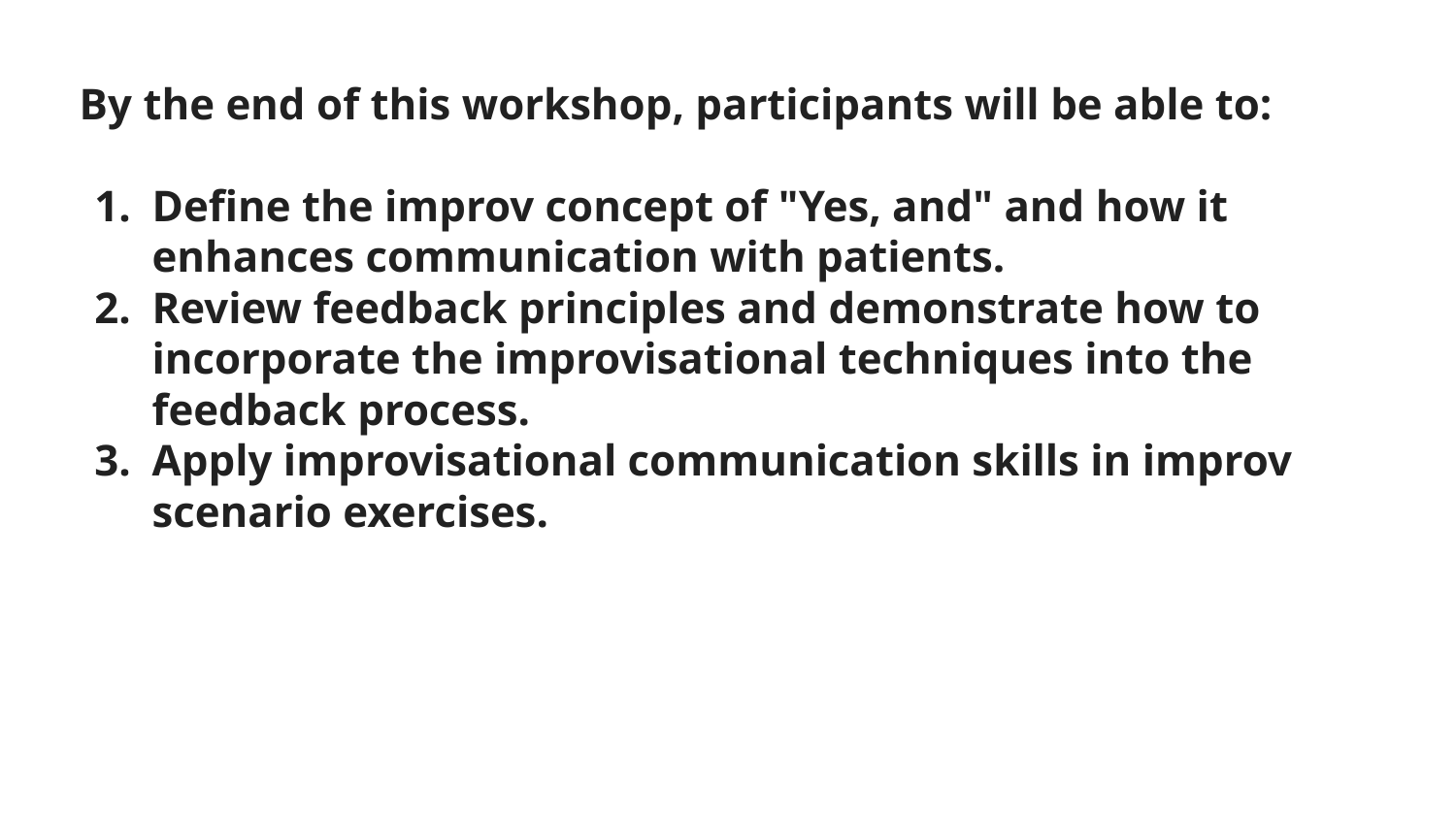

By the end of this workshop, participants will be able to:
Define the improv concept of "Yes, and" and how it enhances communication with patients.
Review feedback principles and demonstrate how to incorporate the improvisational techniques into the feedback process.
Apply improvisational communication skills in improv scenario exercises.

## Slide 4
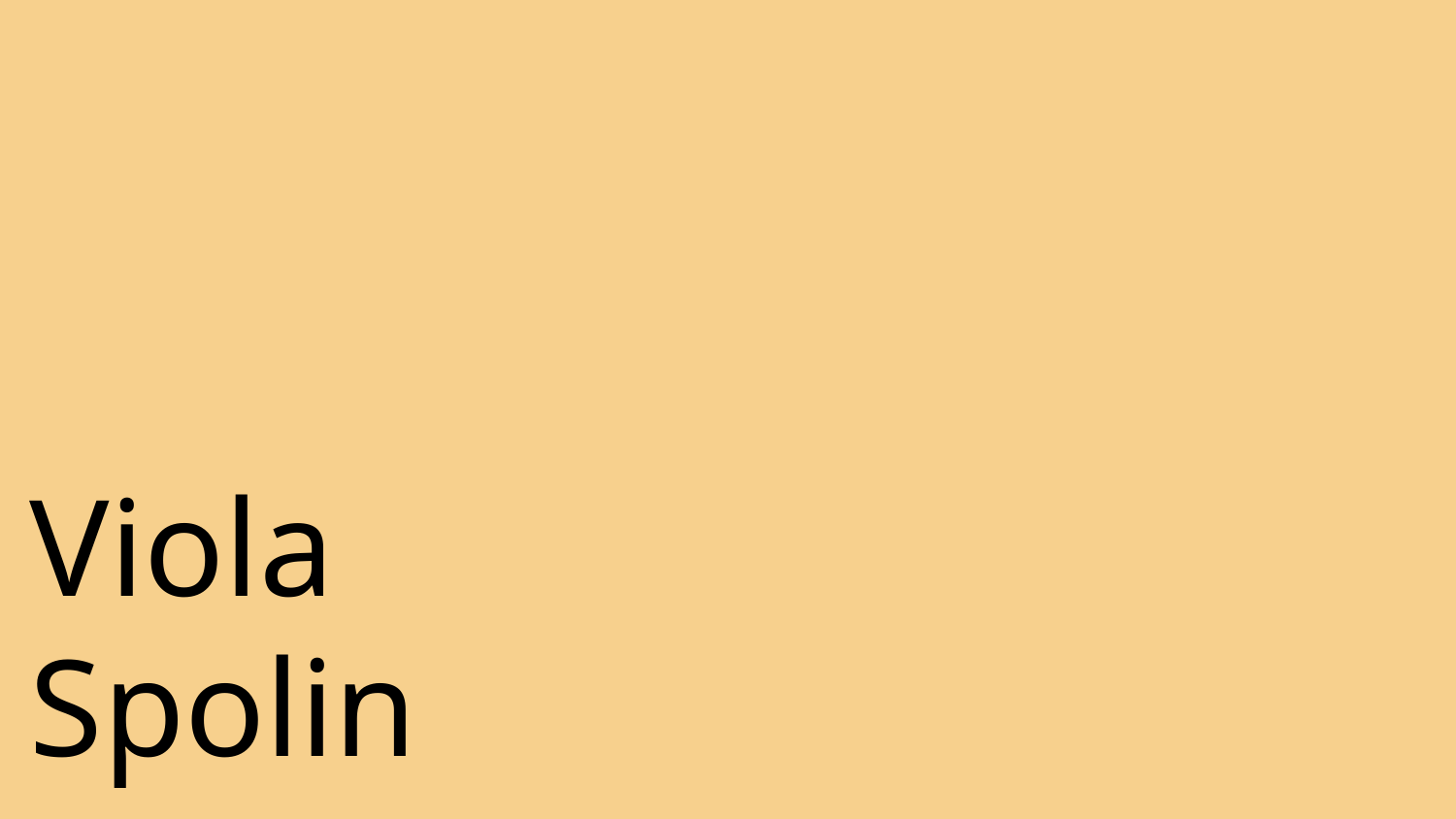

Viola Spolin

## Slide 5
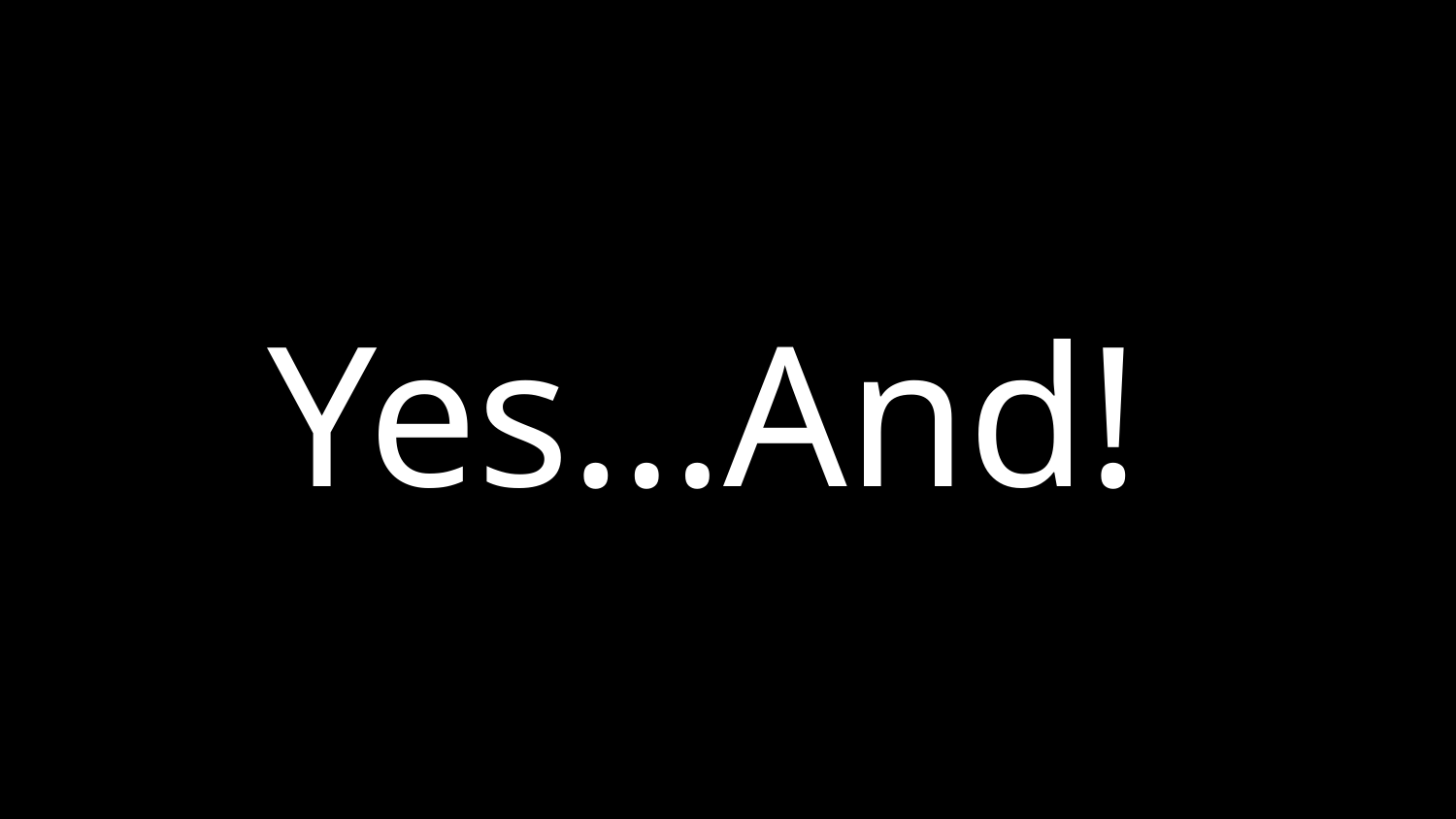

Yes…And!

## Slide 6
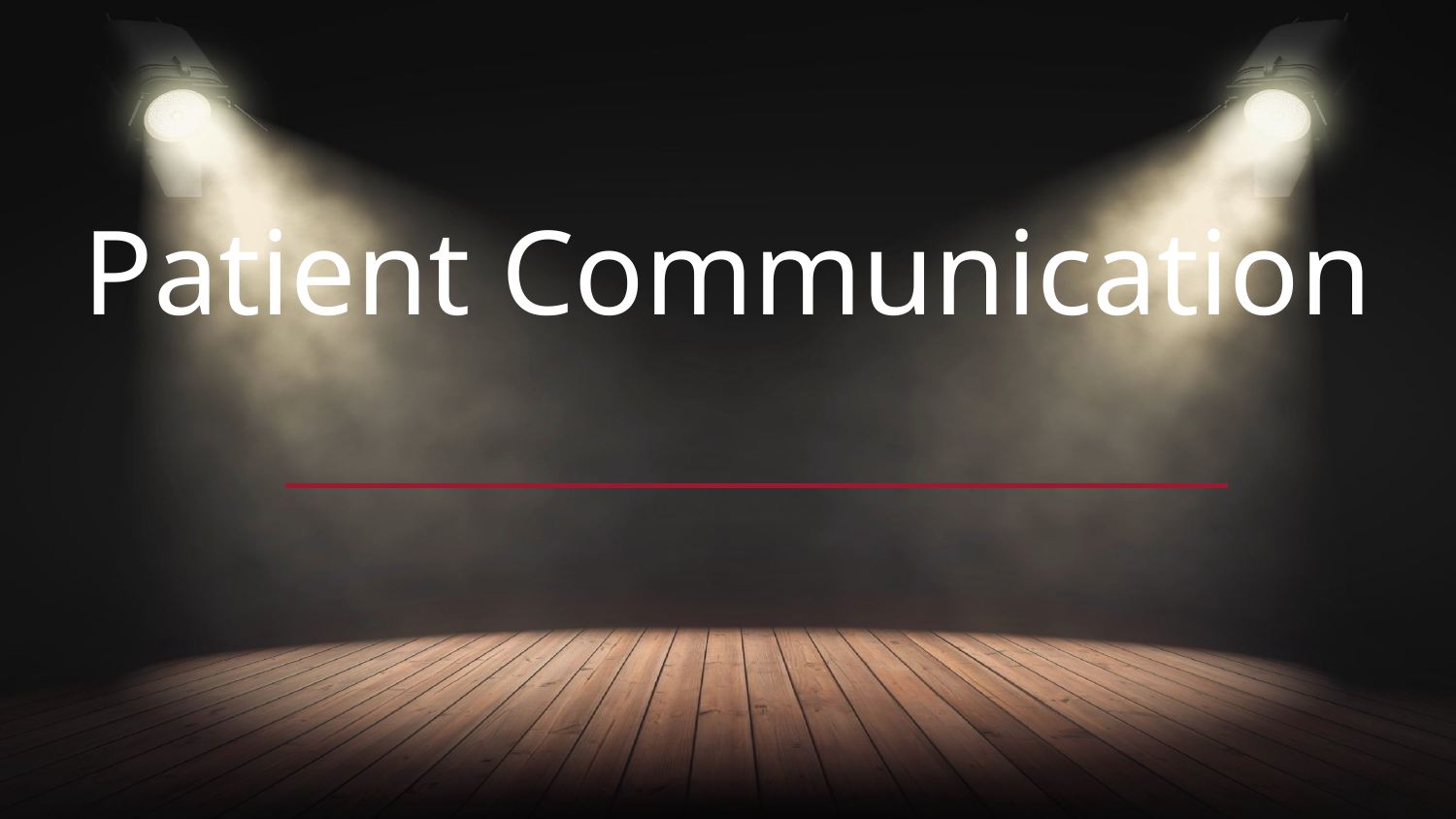

Patient Communication

## Slide 7
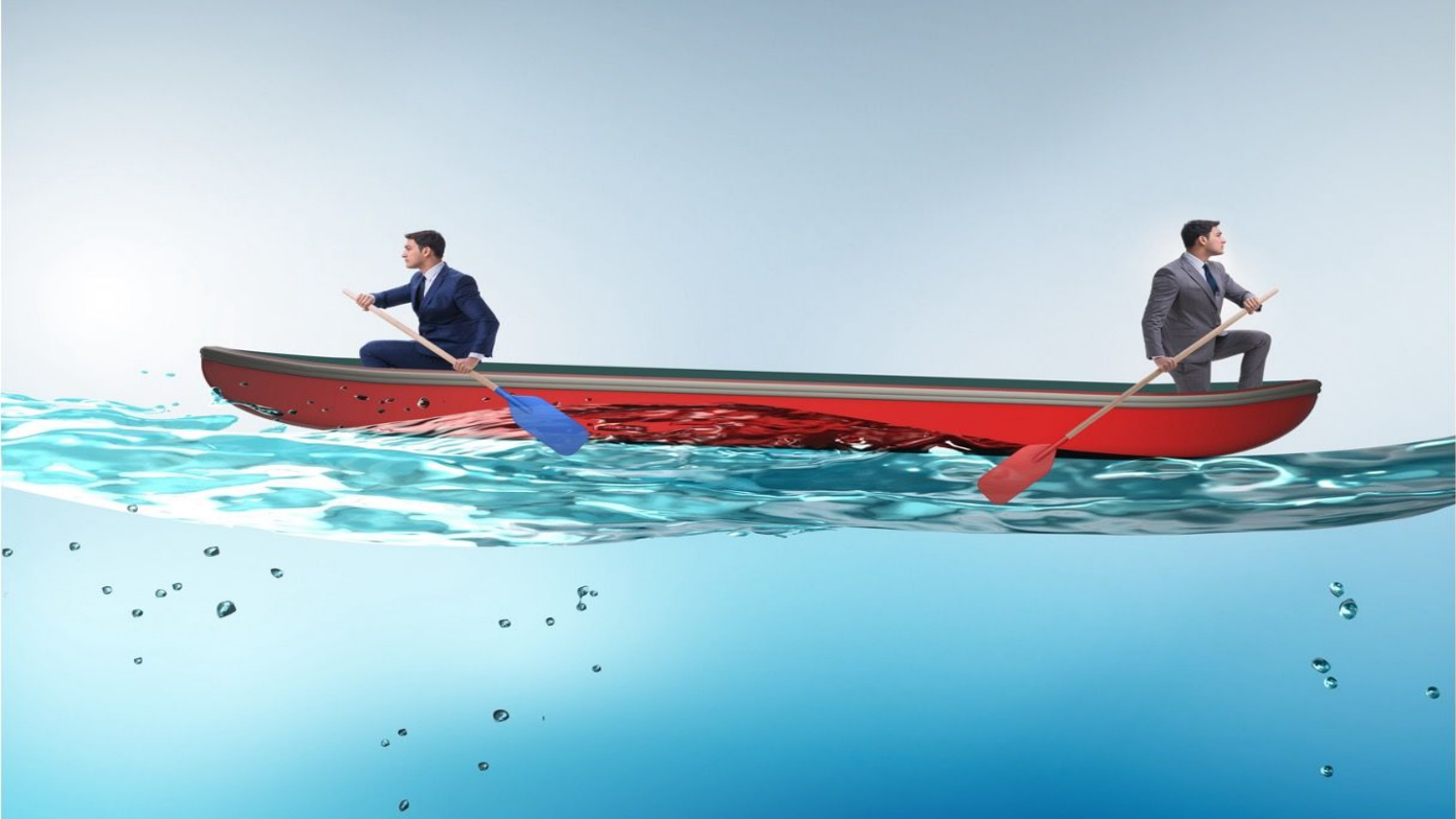

## Slide 8
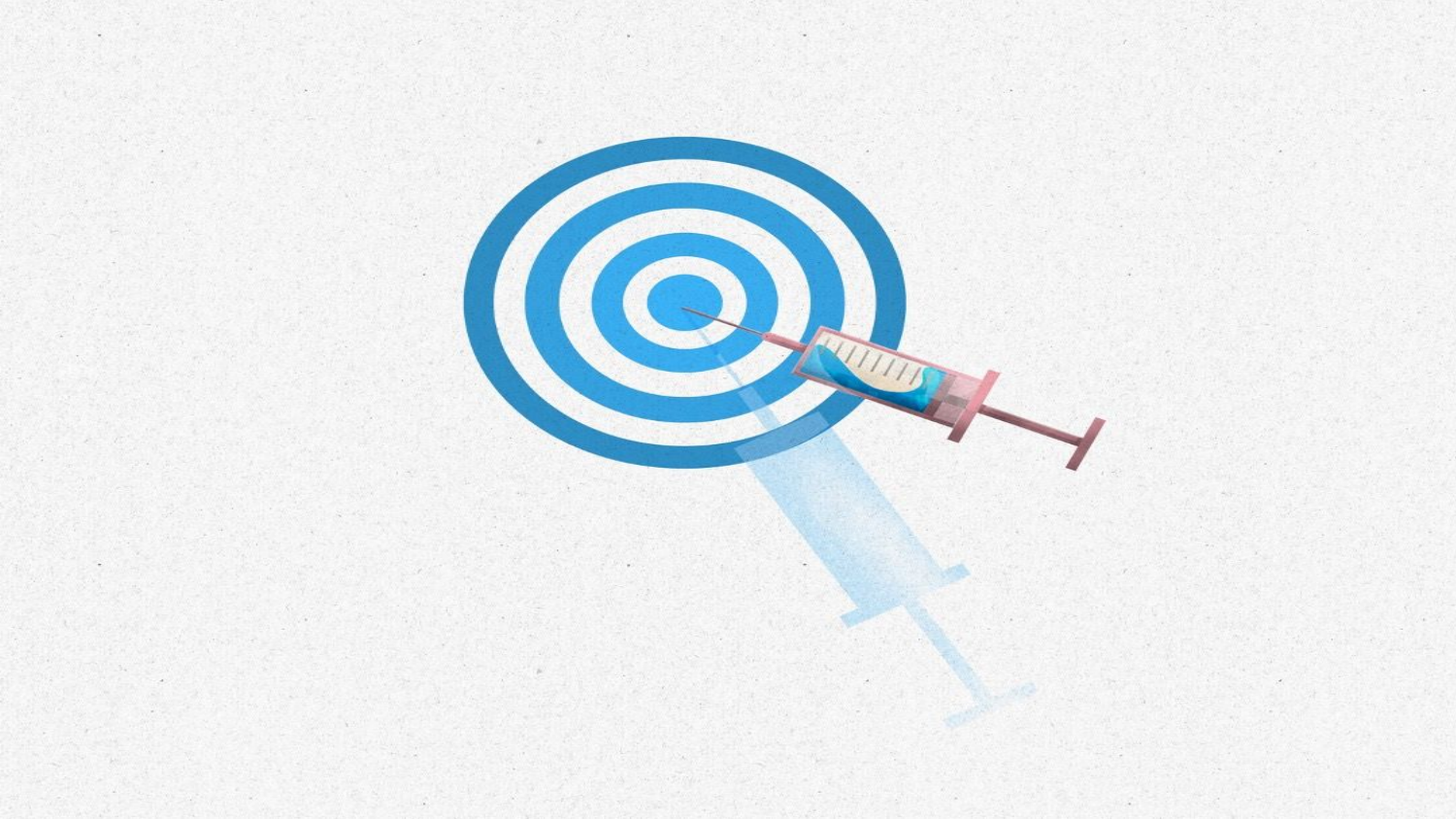

## Slide 9
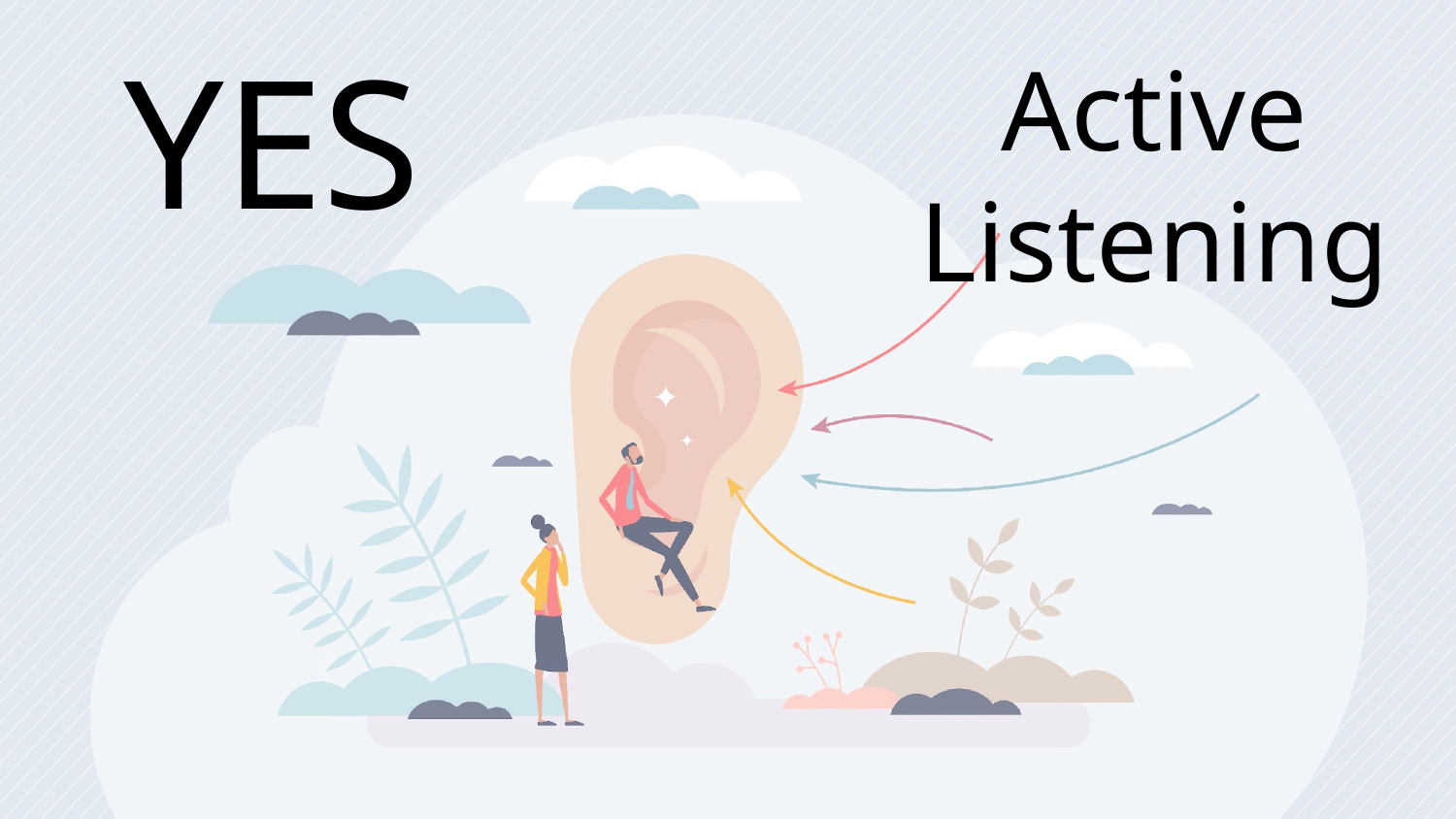

Active Listening
YES

## Slide 10
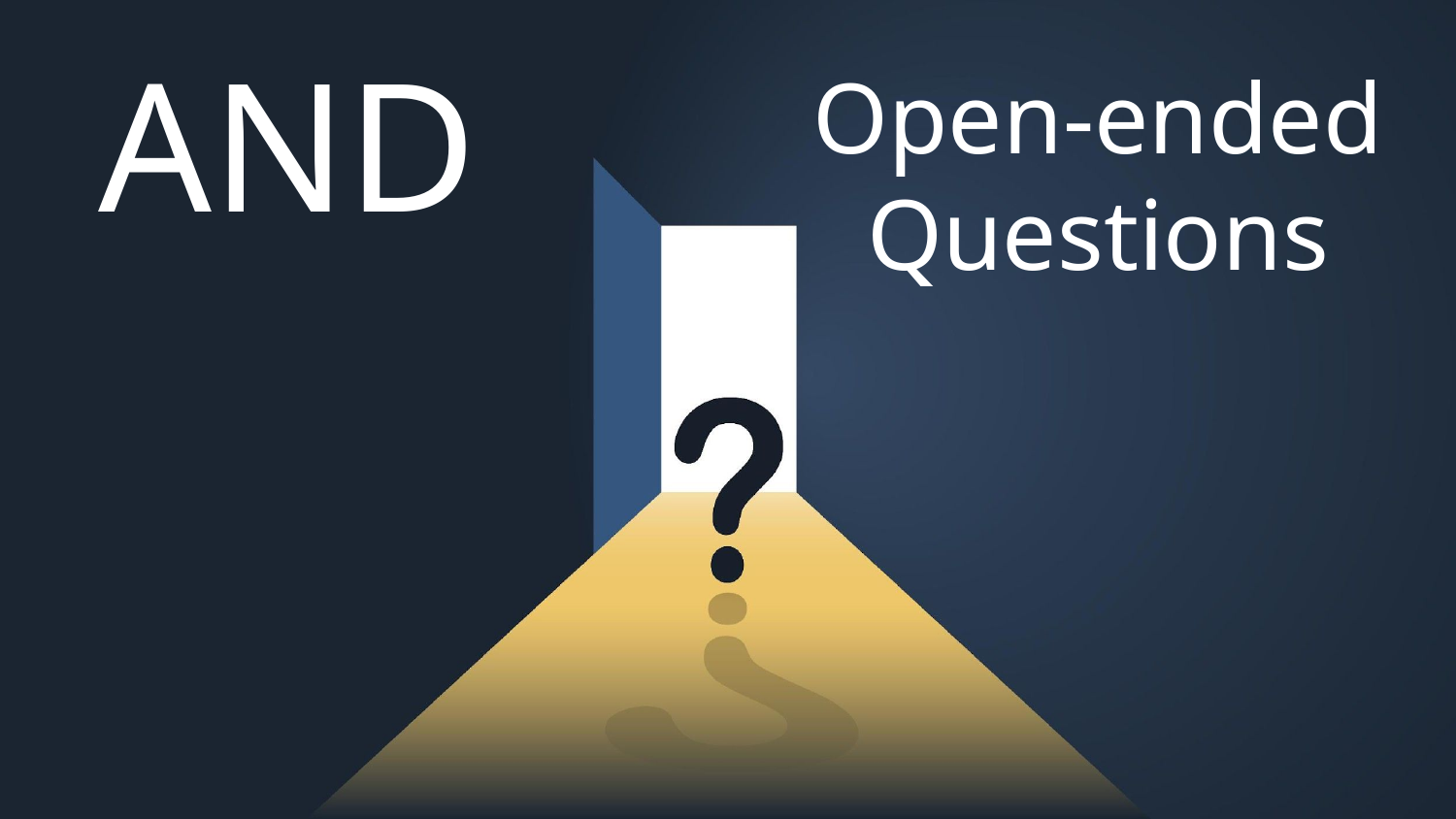

Open-ended Questions
AND

## Slide 11
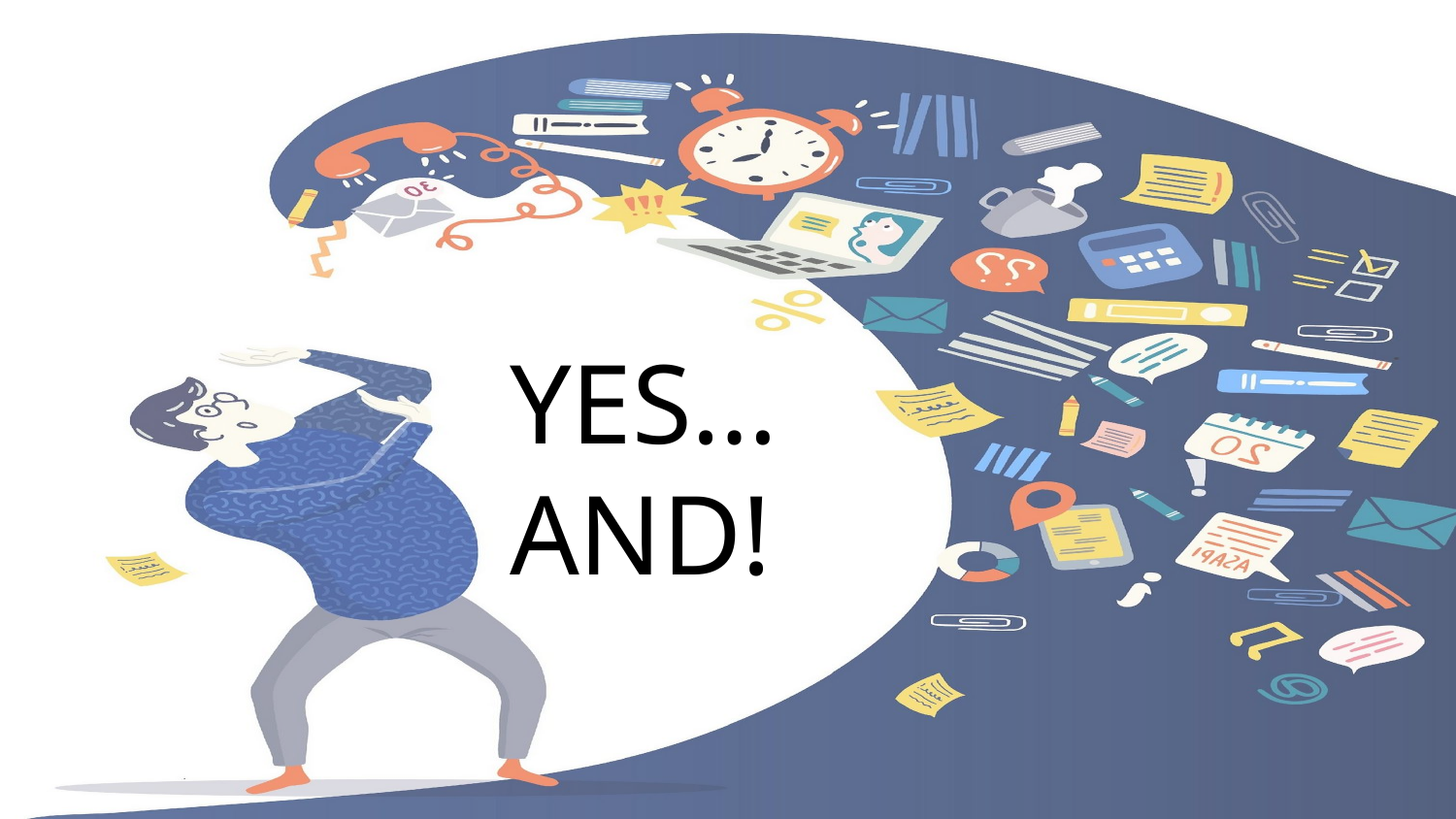

YES…
AND!

## Slide 12
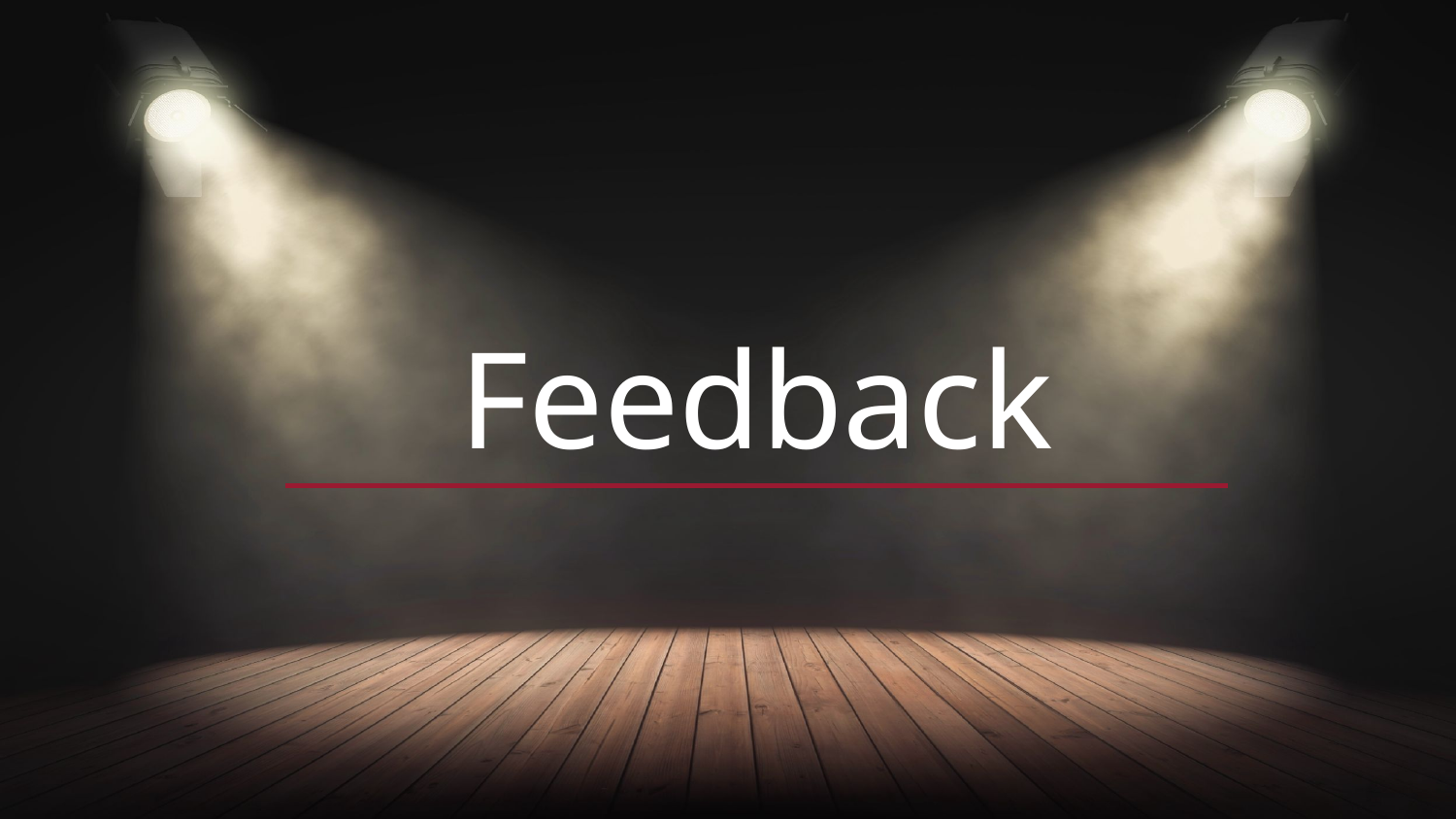

Feedback

## Slide 13
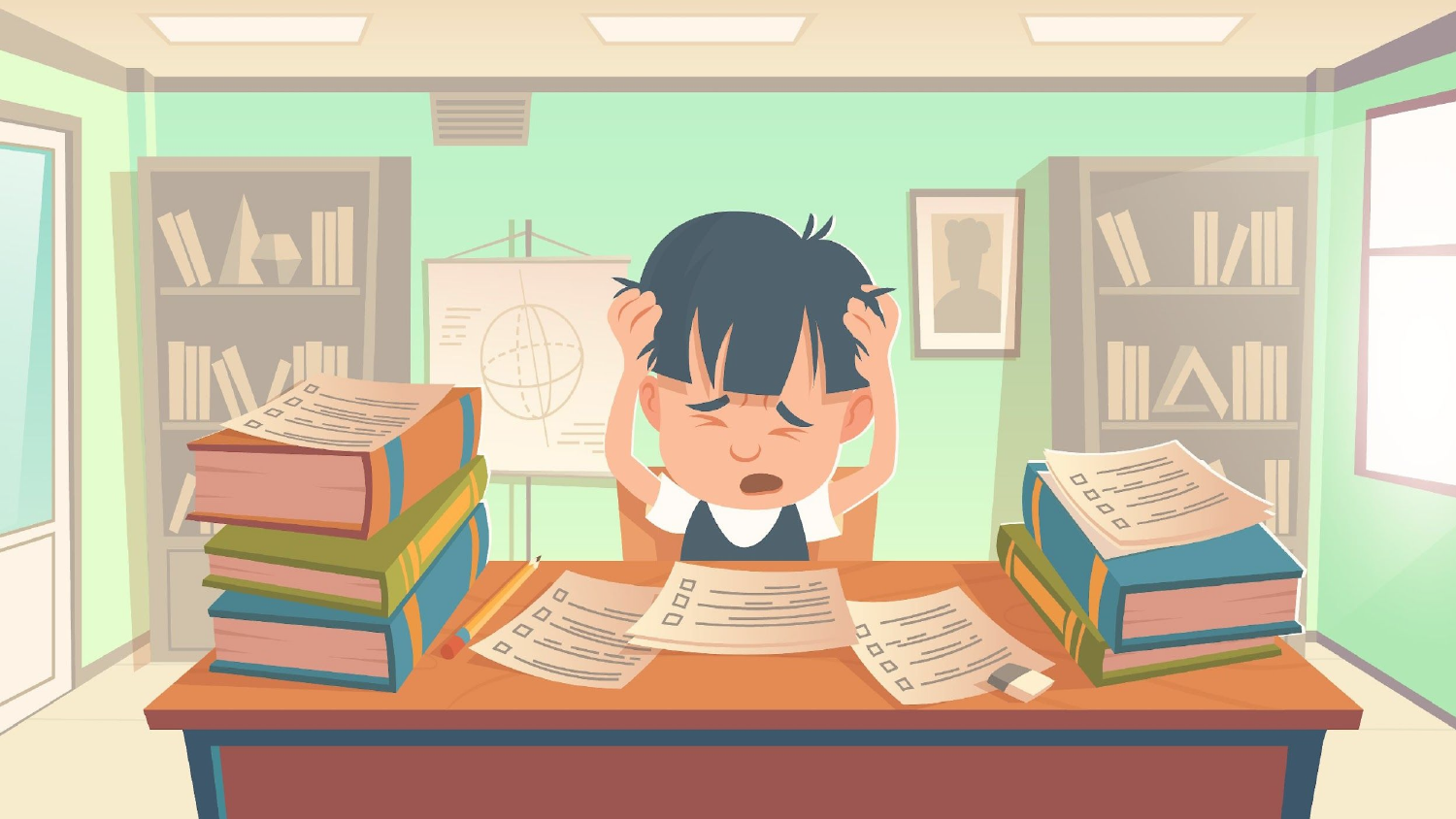

## Slide 14
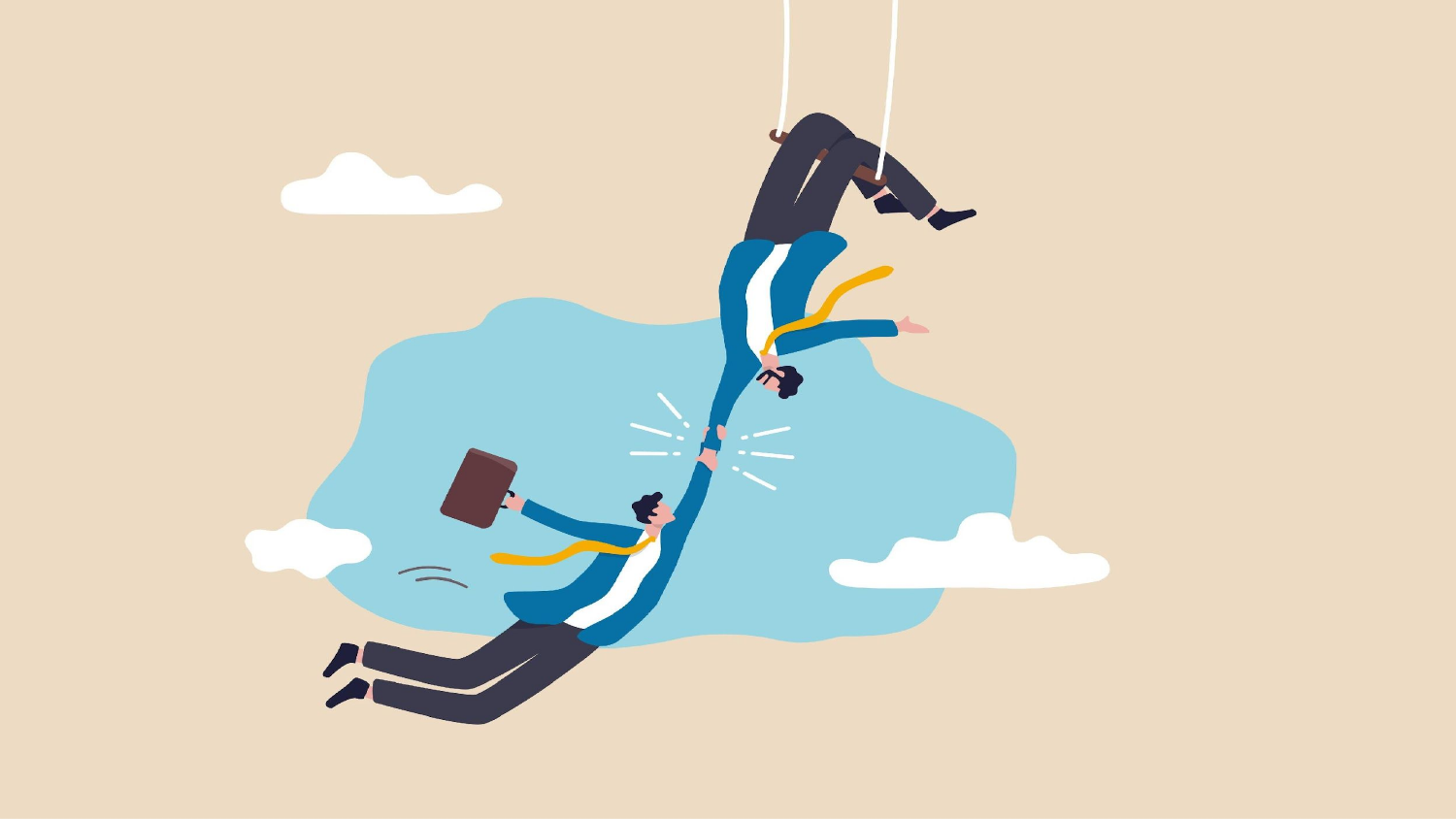

## Slide 15
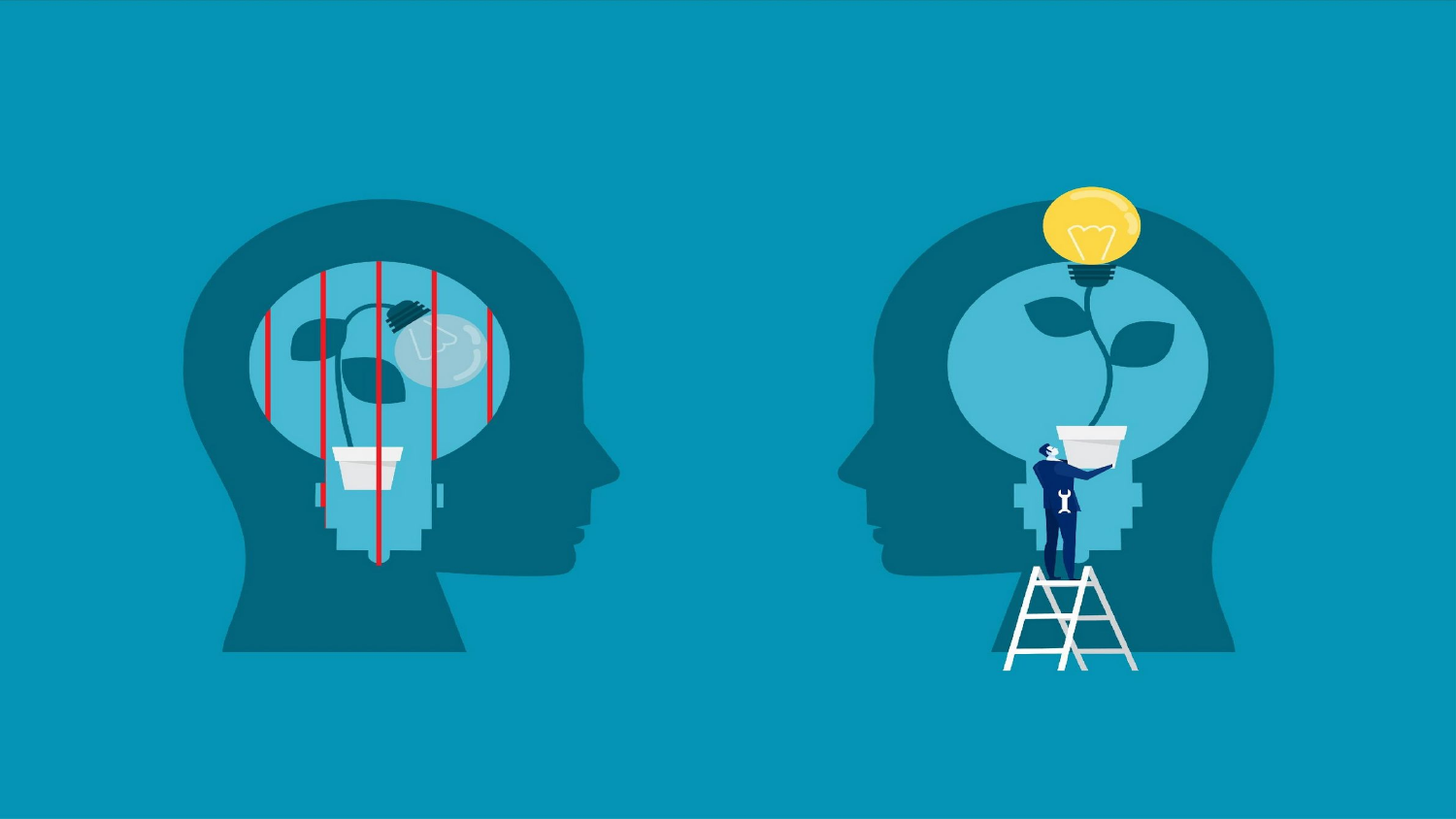

## Slide 16
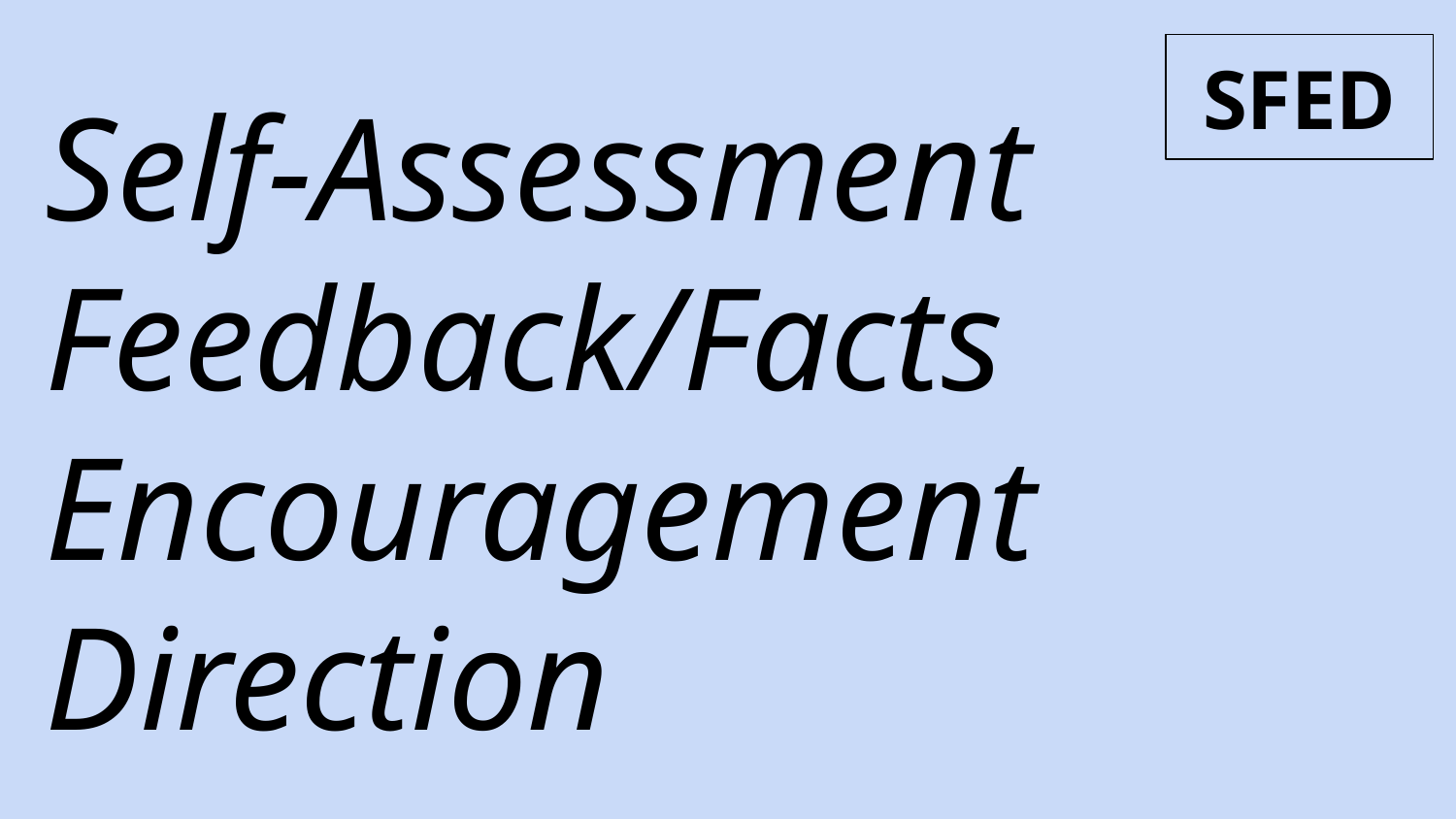

SFED
Self-Assessment
Feedback/Facts
Encouragement
Direction

## Slide 17
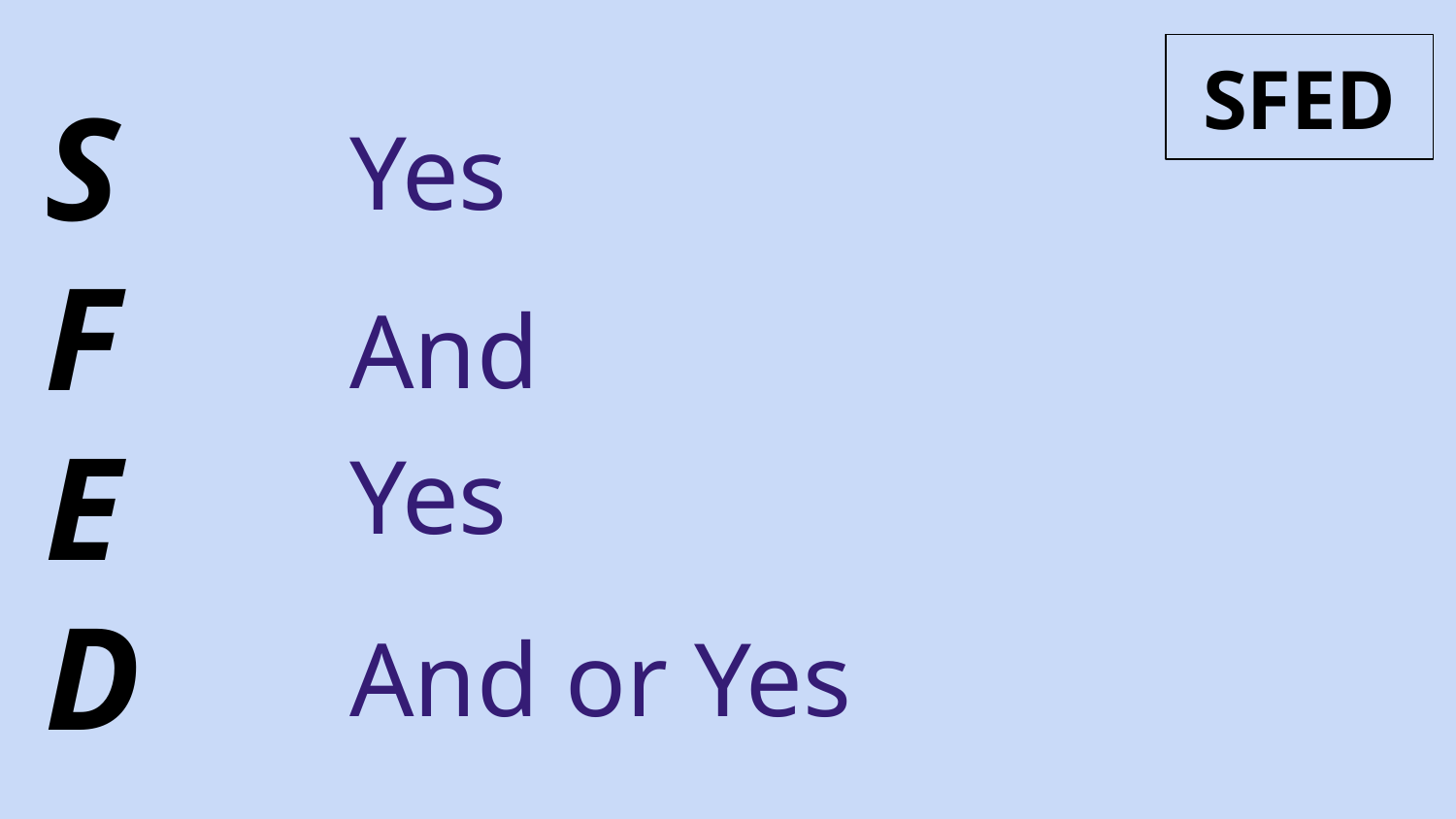

SFED
S
F
E
D
Yes
And
Yes
And or Yes

## Slide 18
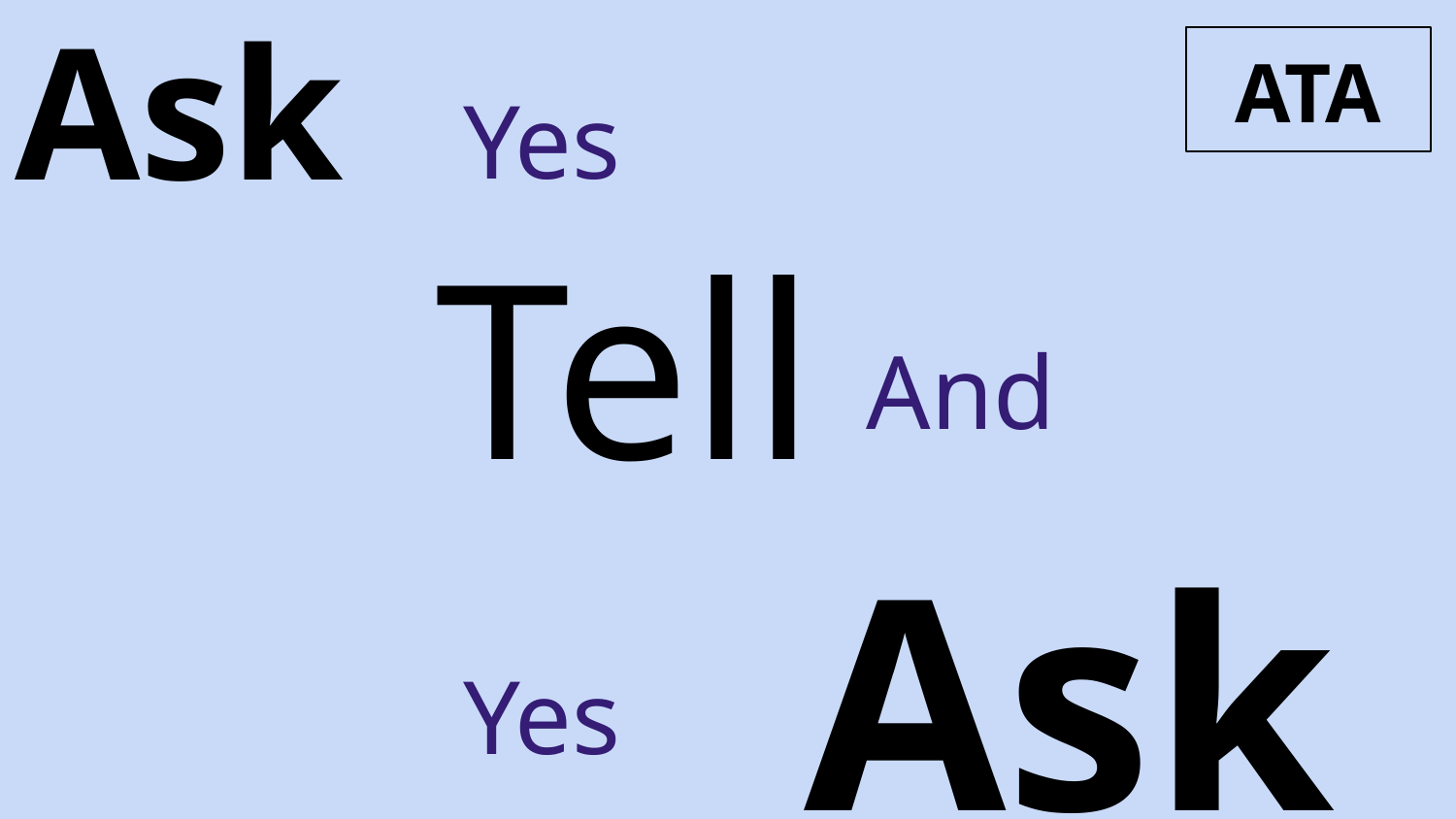

Ask
 Tell
 Ask
ATA
Yes
And
Yes

## Slide 19
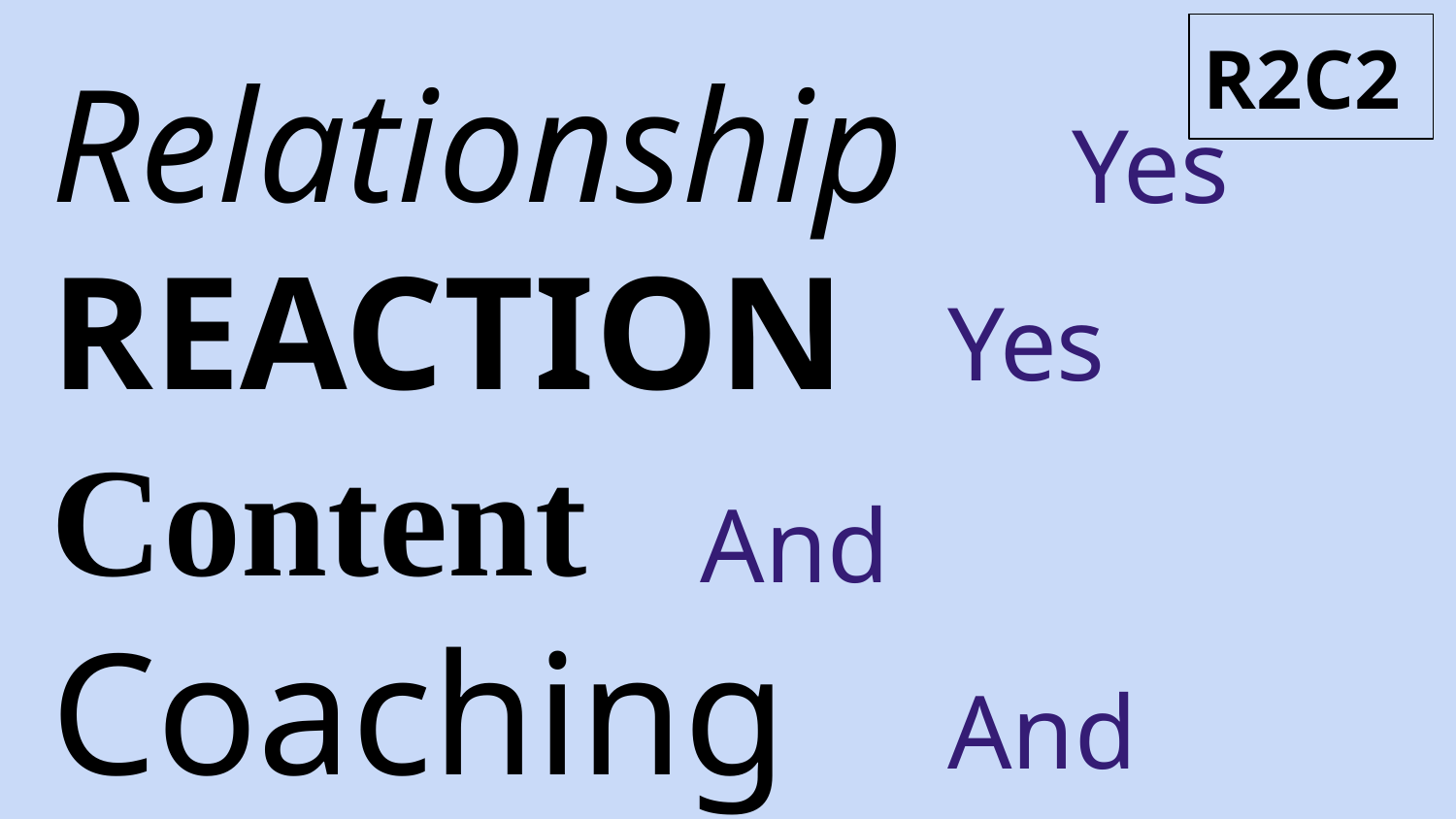

R2C2
Relationship
REACTION
Content
Coaching
Yes
Yes
And
And

## Slide 20
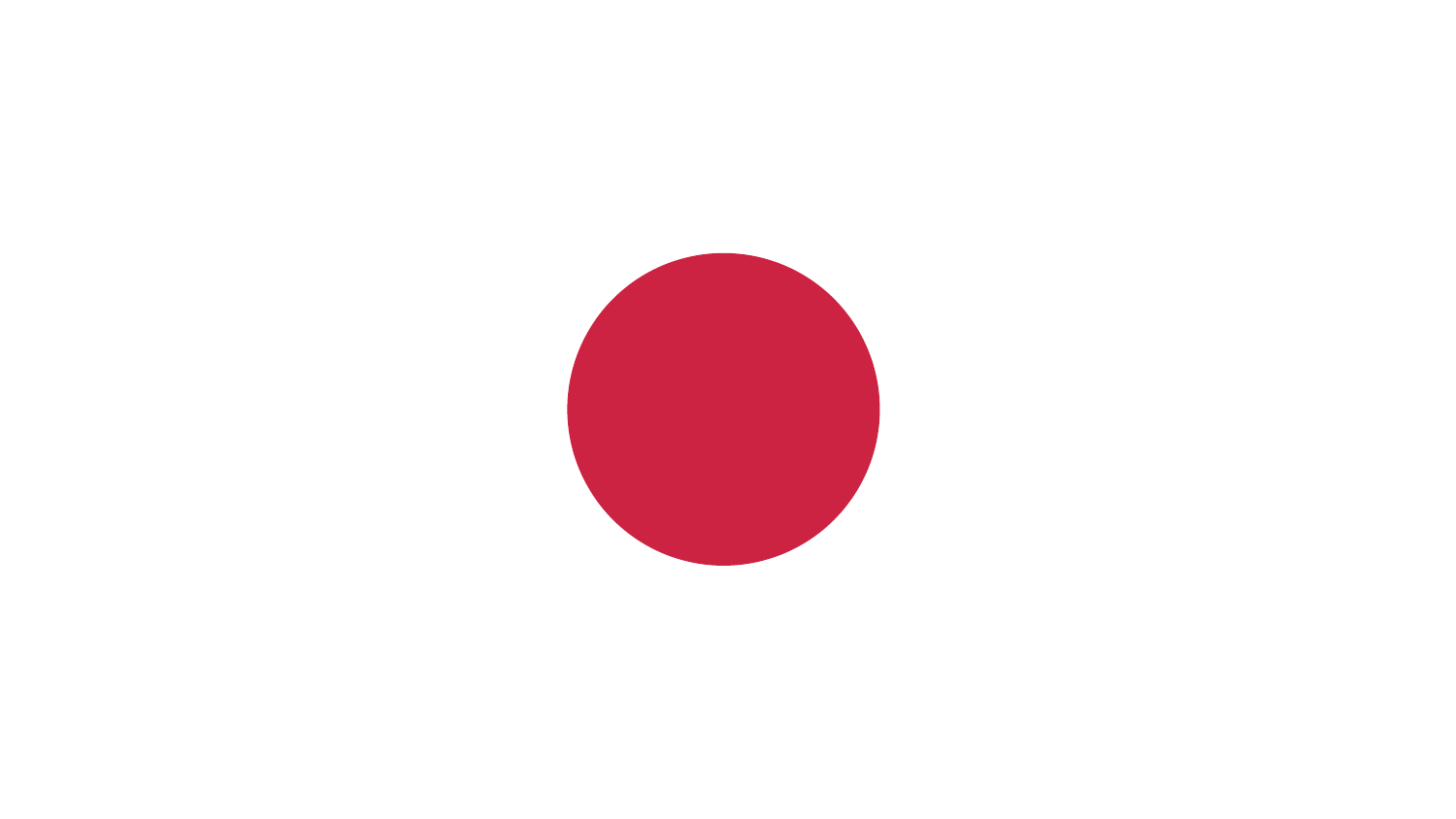

## Slide 21
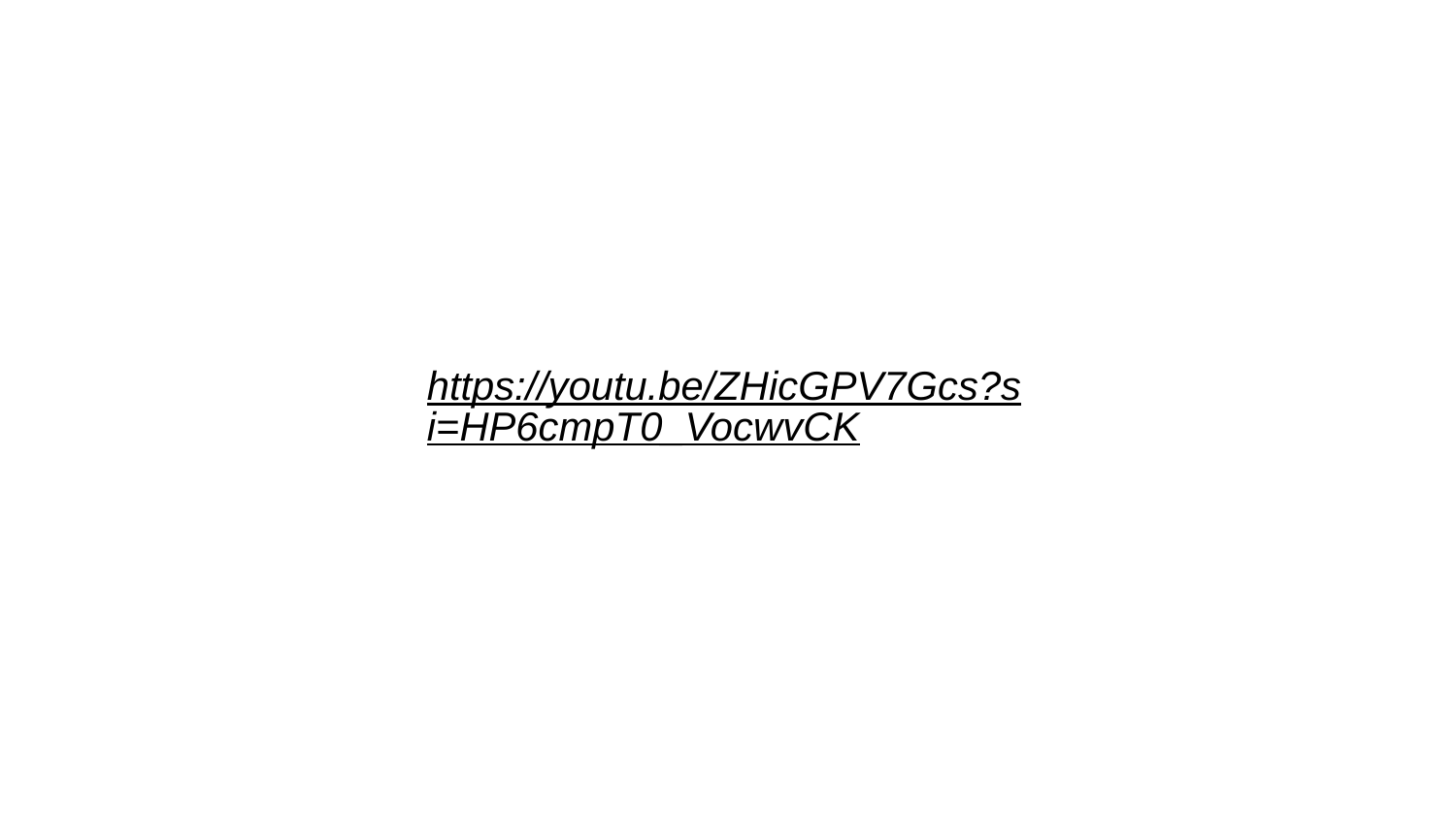

https://youtu.be/ZHicGPV7Gcs?si=HP6cmpT0_VocwvCK

## Slide 22
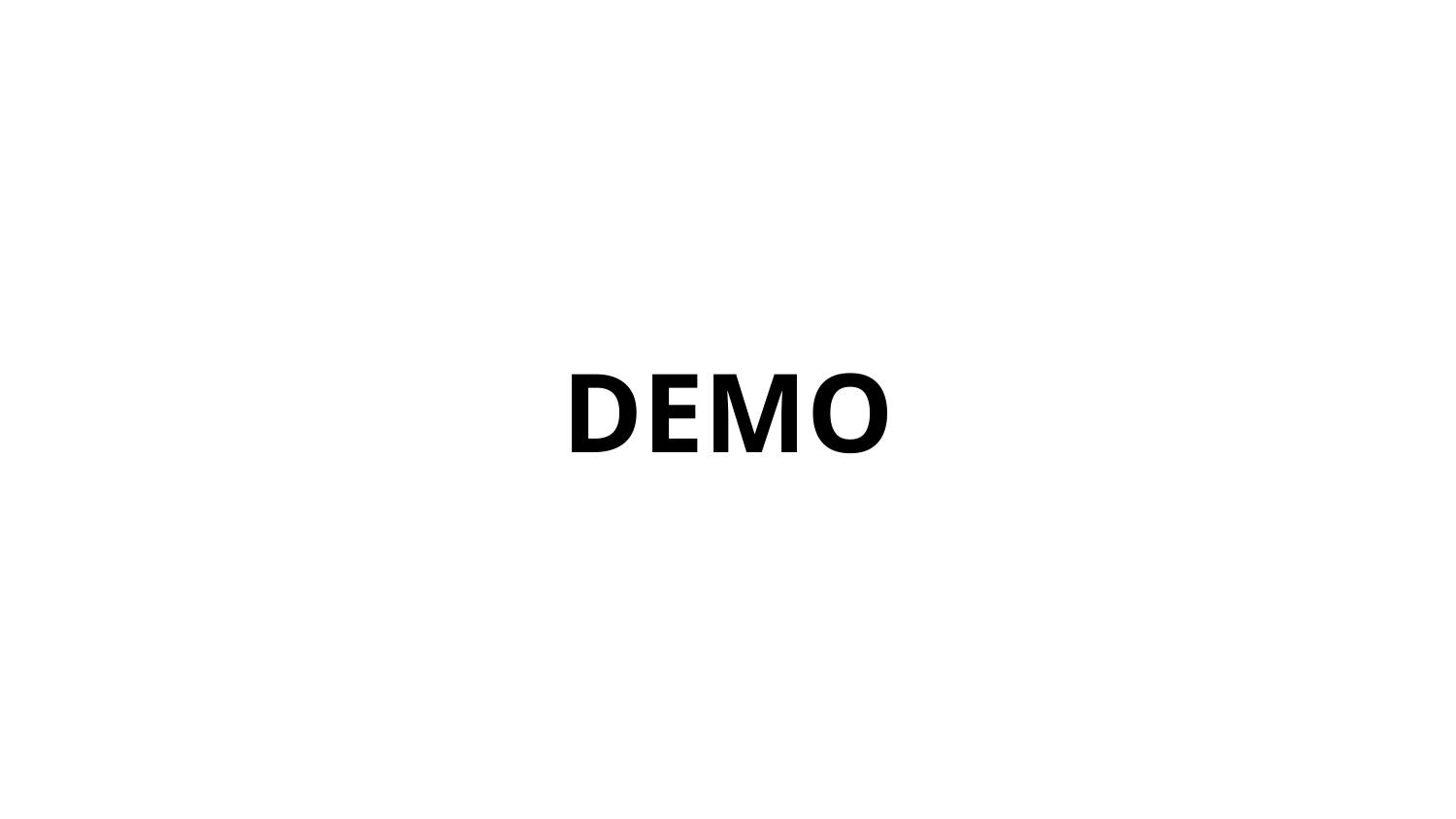

DEMO

## Slide 23
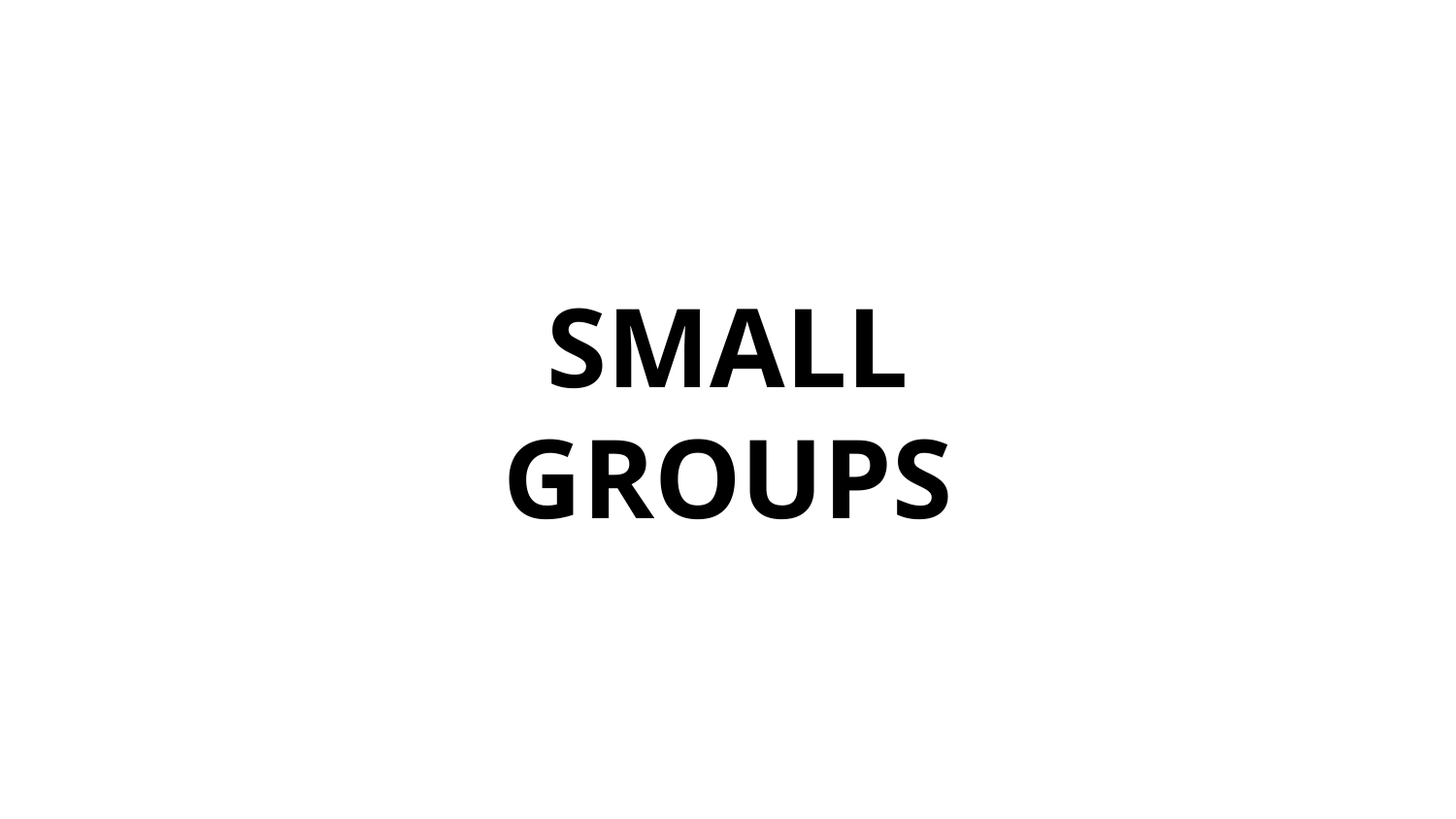

SMALL GROUPS

## Slide 24
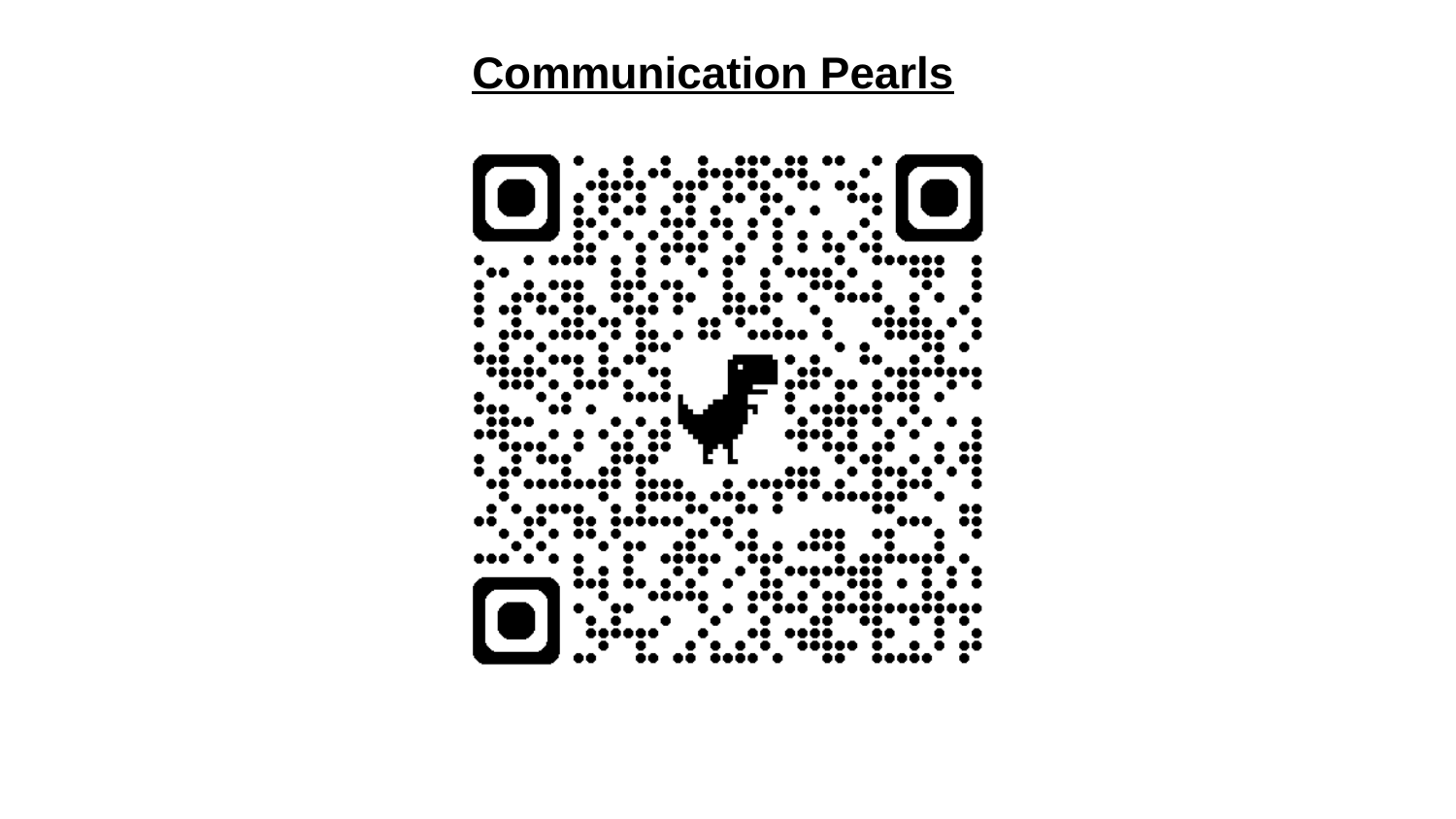

Communication Pearls

## Slide 25
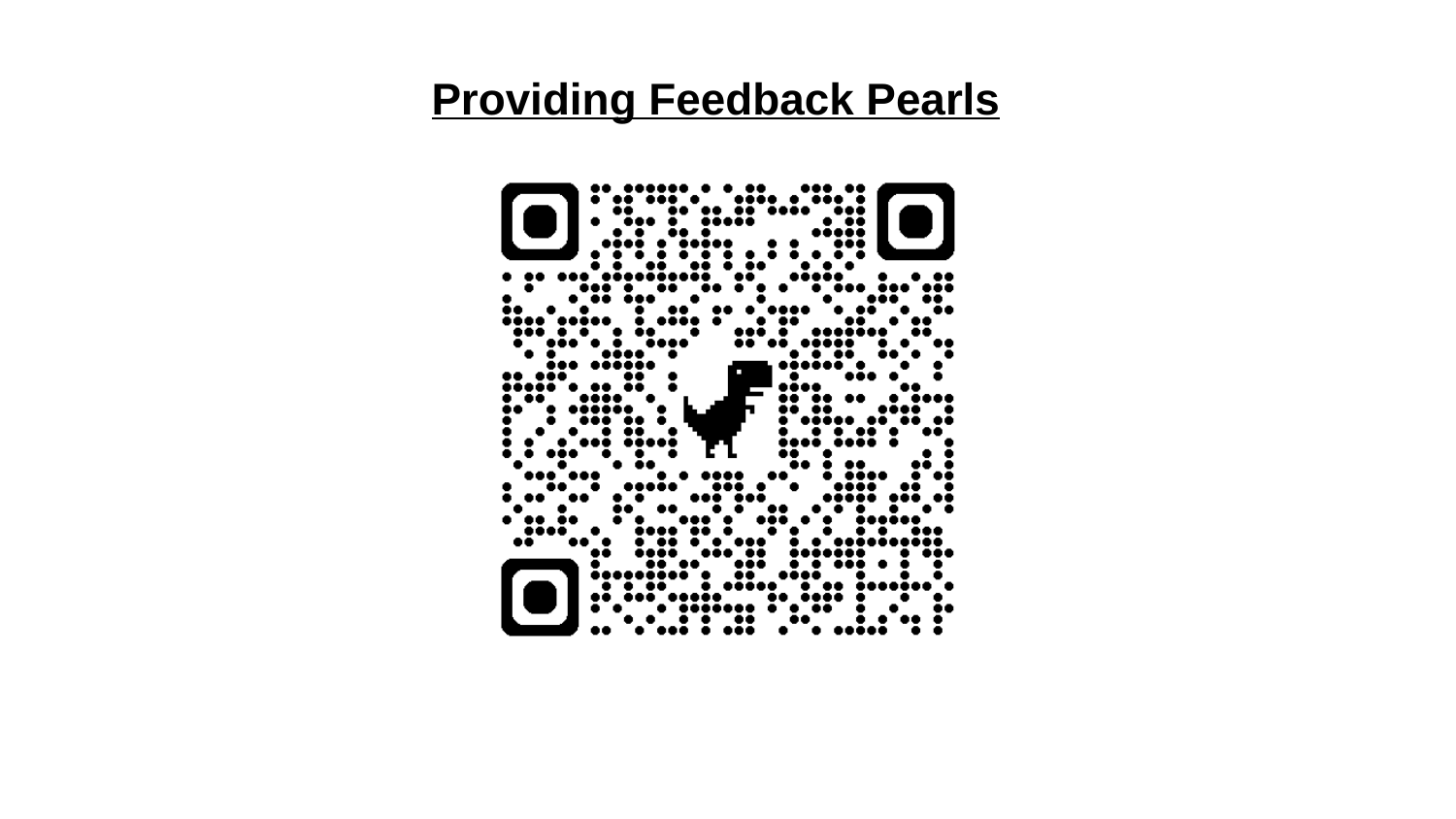

Providing Feedback Pearls

## Slide 26
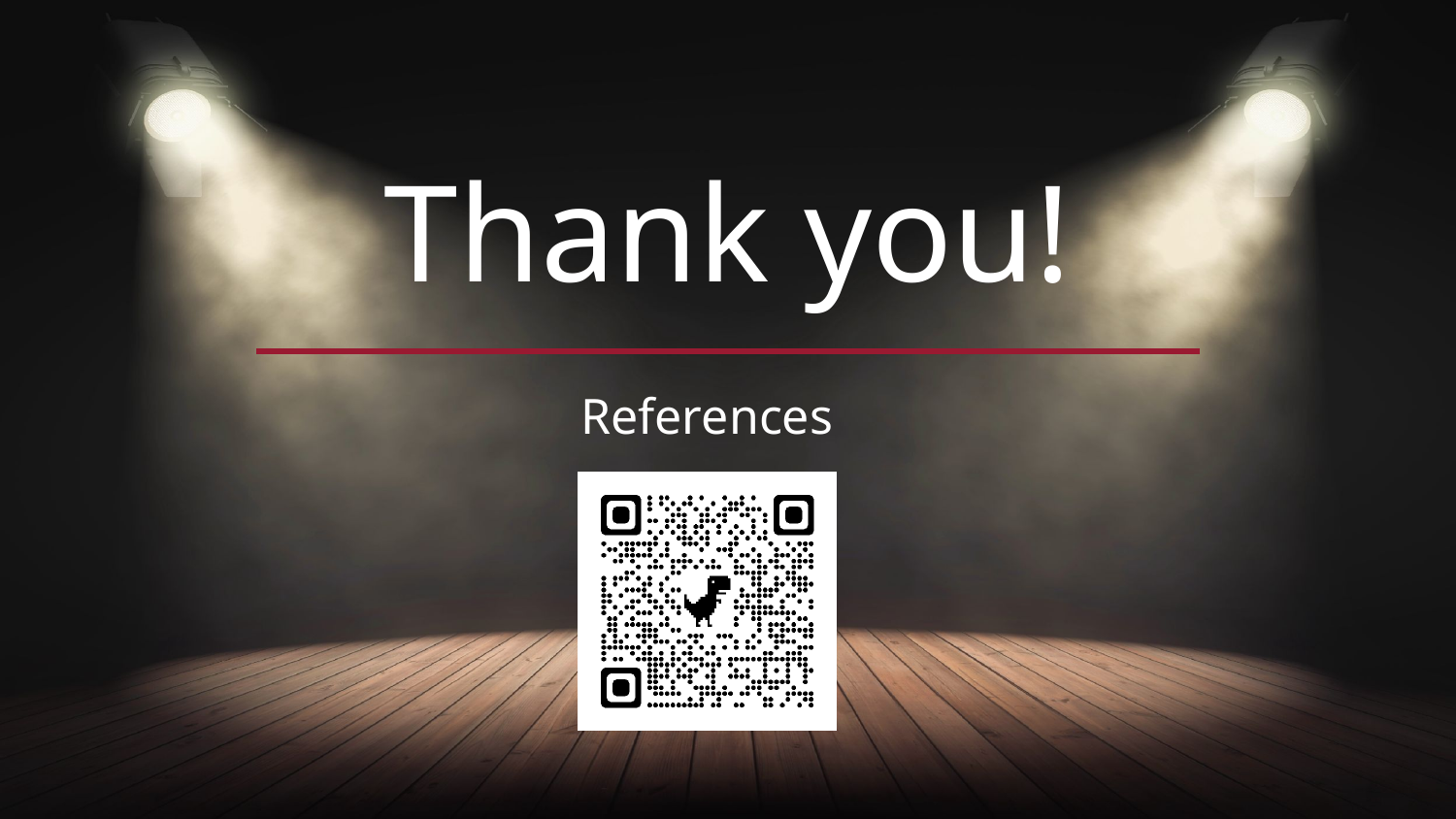

Thank you!
References
